# Jaw anatomy of *Potamogale velox* (Tenrecidae, Afrotheria) with a focus on cranial arteries and the coronoid canal in mammals

**DOI:** 10.7717/peerj.1906

**Published:** 2016-04-12

**Authors:** Robert J. Brocklehurst, Nick Crumpton, Evie Button, Robert J. Asher

**Affiliations:** 1 Faculty of Life Sciences, University of Manchester, Manchester, United Kingdom; 2 Department of Zoology, University of Cambridge, Cambridge, United Kingdom; 3 Research Department of Cell & Developmental Biology, University College London, United Kingdom

**Keywords:** Afrotheria, Ontogeny, Arteries, Mandible, Coronoid canal, Paenungulata, Soft tissues

## Abstract

Afrotheria is a strongly supported clade within placental mammals, but morphological synapomorphies for the entire group have only recently come to light. Soft tissue characters represent an underutilized source of data for phylogenetic analysis, but nonetheless provide features shared by some or all members of Afrotheria. Here, we investigate the developmental anatomy of *Potamogale velox* (Tenrecidae) with histological and computerized tomographic data at different ontogenetic ages, combined with osteological data from other mammals, to investigate patterns of cranial arterial supply and the distribution of the coronoid canal. *Potamogale* is atypical among placental mammals in exhibiting a small superior stapedial artery, a primary supply of the posterior auricular by the posterior stapedial artery, and the development of vascular plexuses (possibly with relevance for heat exchange) in the posterior and dorsal regions of its neck. In addition, the posterior aspect of Meckel’s cartilage increases its medial deflection in larger embryonic specimens as the mandibular condyle extends mediolaterally during embryogenesis. We also map the distribution of the coronoid canal across mammals, and discuss potential confusion of this feature with alveoli of the posterior teeth. The widespread distribution of the coronoid canal among living and fossil proboscideans, sirenians, and hyracoids supports previous interpretations that a patent coronoid canal is a synapomorphy of paenungulates, but not afrotherians as a whole.

## Introduction

The Tenrecidae (tenrecs) is one of two insectivoran-grade taxa in Afrotheria, along with the Chrysochloridae (golden moles). A third afrotherian group (macroscelideans, i.e., sengis or elephant shrews) mixes features present in both “insectivoran” and “ungulate” grade mammals (see reviews by Asher (2005) and Asher & Seiffert (2010)). Until the 1990s, tenrecids and chrysochlorids were generally considered to have a close evolutionary relationship with hedgehogs, shrews, moles, and *Solenodon* in the Lipotyphla (Haeckel, 1866; Nowak, 1999). For many years, the “Lipotyphla” as defined by Haeckel (1866) was thought to represent a natural, monophyletic group, united by six main morphological synapomorphies: the expansion of the maxilla into the orbital mosaic, a simplified gut without a cecum, reduction of the pubic symphysis, a mobile proboscis, an incomplete zygomatic arch, and a hemochorial placenta (Butler, 1972; Butler, 1988). Other investigators noted the tenuous basis for including tenrecs and golden moles in Lipotyphla, and recognized their possible phylogenetic relationships elsewhere among mammals (Broom, 1916; MacPhee & Novacek, 1993). In particular, MacPhee & Novacek (1993) found that none of the six main characters proposed as synapomorphies by Butler (1972) uniquely defined the group to the exclusion of other placental mammals.

A large body of genetic evidence (e.g., Meredith et al., 2011; see reviews in Tabuce, Asher & Lehmann (2008) and Asher & Seiffert (2010)) now unambiguously supports tenrecs and golden moles as members of Afrotheria, also including the Tubulidentata (aardvarks), Macroscelididae (sengis), Proboscidea (elephants), Hyracoidea (hyraxes), and Sirenia (sea cows). With the benefit of a now well-corroborated tree documenting how placental orders are interrelated, several investigators have discovered anatomical features that represent afrotherian synapomorphies (Tabuce, Asher & Lehmann, 2008; Asher & Olbricht, 2009; Asher & Seiffert, 2010). These include both hard (Sánchez-Villagra, Narita & Kuratani, 2007; Tabuce et al., 2007; Seiffert, 2007; Asher & Lehmann, 2008) and soft tissue (Werdelin & Nilsonne, 1999; Mess & Carter, 2006; Carter et al., 2006) characters. The latter are typically difficult to collect due to inaccessibility of materials and necessity of invasive sampling, although the diversity of certain soft tissue regions is becoming increasingly well known (e.g., Wible, 1986; Wible, 1987; Mossman, 1987; Stephan, Baron & Frahm, 1991; Carter et al., 2006; Diogo & Abdala, 2010; Cox & Jeffery, 2011). Despite these exceptions, comparative anatomical data on placentation, vasculature, internal organs, and myology tend to be less widely known across mammals compared to bones and teeth.

**Figure 1:**
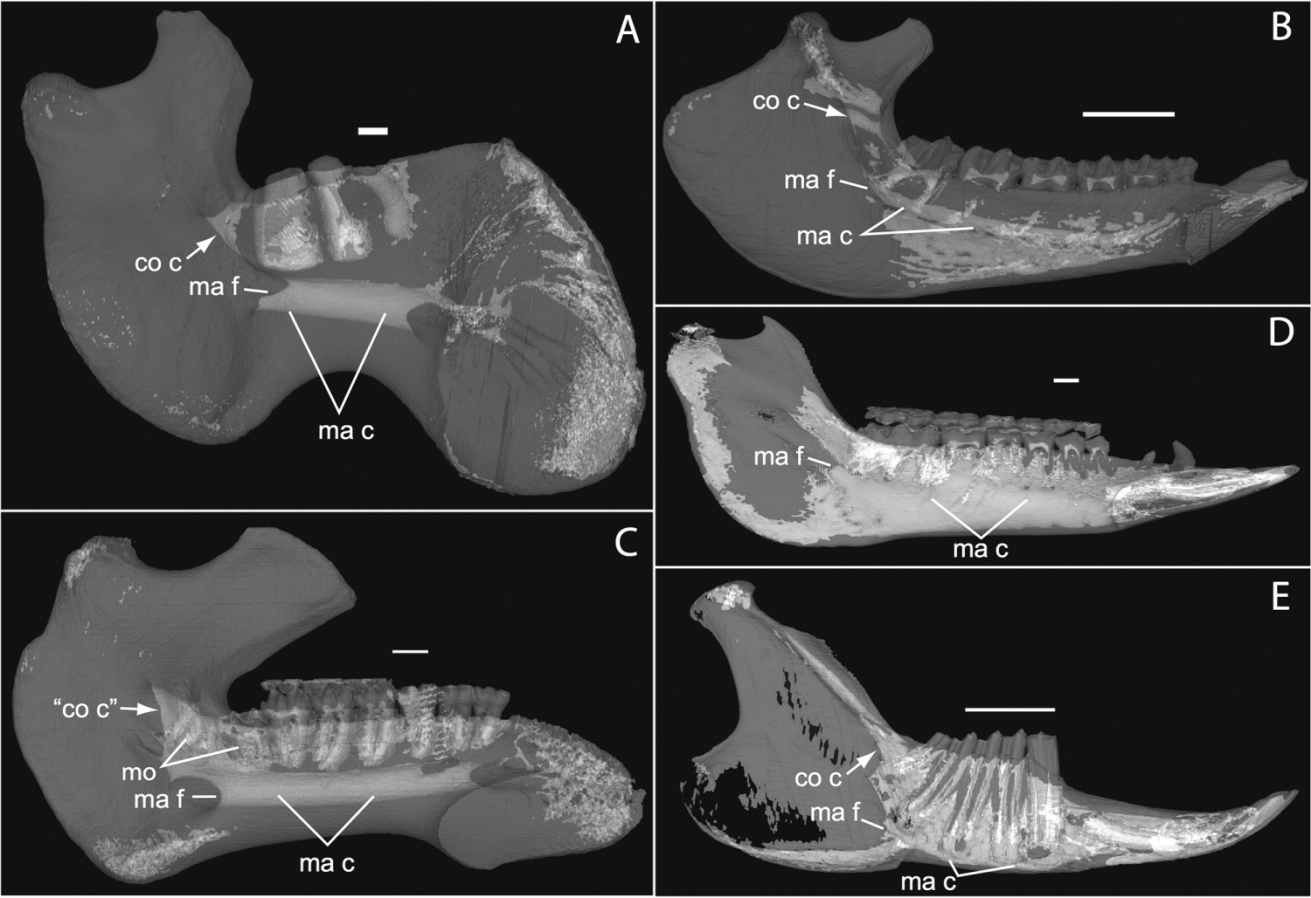
Micro-CT scanned jaws of (A) *Dugong dugon* (UMZC 2016-2), (B) *Procavia capensis* (TMM M4351), (C) *Trichechus senegalensis* (AMNH 53939), (D) *Sus scrofa* (TMM M454), (E) *Lepus californicus* (TMM M7500). Light areas represent sinuses, canals, and other internally open regions. For scanning details see Table 2; for abbreviations see Table 3. Scale bars indicate approximately 1 cm.

Here, we describe the ontogeny of cranial arteries in the insectivoran-grade afrothere *Potamogale* and document the hard- and soft-tissue relations of another morphological feature found in some afrotheres: the coronoid canal (Tabuce et al., 2012). The coronoid canal has been suggested to be a potential synapomorphy of the Paenungulata (i.e., elephant, sea cow, hyrax; see Tassy & Shoshani, 1988; Kondrashov, 1998; Gheerbrant, Domning & Tassy, 2005), and has also been observed in the stem macroscelidid *Nementchatherium* (Tabuce et al., 2012). In adults of these taxa, the canal joins the postero-internal aspect of the dentary with a foramen posterior to the last molar (Figs. 1A, 1B and 1E); it may also communicate ventrally with the mandibular canal (Tabuce et al., 2012: fig. 3), but in such cases still opens dorsally via a foramen posterior to and separate from dental alveoli. We document the anatomy of this region, including vasculature and osteology, in a developmental series of histologically prepared *Potamogale velox*, digitized and reconstructed in 3D, supplemented by data on cranial arterial supply from the literature (Presley, 1979; Wible, 1987; Asher, 2001; Schunke & Zeller, 2010) and micro-computerized tomographic (μCT) scans of mammalian jaws. Our work expands on previous literature outlining the relevance of cranial anatomy to mammalian systematics in general (Bugge, 1972; Presley, 1979; Cartmill & MacPhee, 1980; MacPhee, 1981; Zeller, 1986; Wible, 1987; Wible & Zeller, 1994) and afrotherians in particular (Asher, 2001).

## Materials and Methods

We investigated four histologically prepared specimens of differing ontogenetic ages from the Presley Collection at the University Museum of Zoology, Cambridge (UMZC), summarized in Table 1. Individual slices were digitized using a Leica MZ 9.5 transmitted light microscope under low (0.5–1.25×) magnification and a Leica DFC420 digital camera. Images were acquired in TIFF format, then converted to JPG with Photoshop and ImageJ (Rasband, 1997) to reduce size. A number of μCT scans of additional taxa (Table 2) were scanned at the University of Cambridge Biotomography Centre, Helmholtz Zentrum Berlin, or downloaded from www.digimorph.org and saved as slices using ImageJ (Rasband, 1997). These were imported into Mimics version 16 (Mimics Innovation Suite, Materialise) and rendered in 3D, either by segmenting out structures of interest, or with transparency set to “high” in order to display relevant areas of jaw anatomy, such as the mandibular canal, coronoid canal, and tooth alveoli. Abbreviations for anatomical structures and institutions are given in Table 3.

**Table 1:** Histologically prepared specimens of *Potamogale velox* used in this study. Measurements are in millimeters except where noted.

**Catalogue Number**	UMZC 2016.Histo.Po1	UMZC 2016.Histo.Po2	UMZC 2016.Histo.Po3	UMZC 2016.Histo.Po4
**Description**	Head	Whole Body	Head	Head
**Head Length**	13	11	24	16
**Jaw Length**	6.2	4.6	11.8	7.3
**Body Length**	29	24	48	30
**Section Orientation**	Coronal	Sagittal	Coronal	Coronal
**Staining**	Trichrome and Haematoxylin & Eosin on alternate slides	Trichrome and Haematoxylin & Eosin on alternate slides	Trichrome and Haematoxylin & Eosin on alternate slides	Trichrome on all slides
**Specimen Slice Distance (μm)**	10	10	12	10
**Stack Slice Distance (μm)**	30	10	36	30
**Number of Slices in Stacks**	306	368	489	392

**Table 2:** Summary of μCT scanned specimens. See Table 3 for abbreviations. UT specimens and associated metadata are from www.digimorph.org.

Taxon	μCT Facility	Voxel size (mm)	Specimen #	Source
*Amblysomus hottentotus*	CBC	0.0156	NFC 2	Original specimen
*Amblysomus hottentotus*	CBC	0.0144	BMNH 4.6.6.3	Original specimen
*Dugong dugon*	CBC	0.2500	UMZC 2016-2 (Ceylon 5)	Original specimen
*Elephas maximus*	UT	0.2011	TMM M6445	digimorph.org
*Lepus californicus*	UT	0.1439	TMM M7500	digimorph.org
*Microgale dobsoni*	CBC	0.0161	MNHN 1932-3480	Original specimen
*Potamogale velox*	HZB	0.0238	UMZC E5425B	Original specimen
*Potamogale velox*	CBC	0.0606	UMZC E5425H	Original specimen
*Potamogale velox*	CBC	0.0583	UMZC E5425I	Original specimen
*Procavia capensis*	UT	0.0790	TMM M4351	digimorph.org
*Setifer setosus*	CBC	0.0219	MNHN 1882-1566	Original specimen
*Setifer setosus*	CBC	0.0217	MNHN 1962-1064	Original specimen
*Sus scrofa*	UT	0.5	TMM M454	digimorph.org
*Tapirus terrestris*	UT	1.0	TMM M16	digimorph.org
*Trichechus senegalensis*	UT	0.5	AMNH 53939	digimorph.org

**Table 3:** Abbreviations used in the text, tables, and figures.

*Institutional*	
AMNH	American Museum of Natural History New York, USA
BMNH	Natural History Museum London, UK
CBC	Cambridge Biotomography Centre, UK
HZB	Helmholtz Zentrum Berlin, Germany
MNHN	Museum Nacional d’Histoire Naturelle Paris, France
NFC	Personal collection RJ Asher, UK
TMM	Texas Memorial Museum Austin, USA
UMZC	University Museum of Zoology Cambridge, UK
UT	University of Texas at Austin, USA
*Anatomical*	
ap	ascending pharyngeal artery
ba	basilar artery
c	lower canine
cc	common carotid artery
co c	coronoid canal
co f	coronoid fossa
cp	cribriform plate
csn	carotid sympathetic nerve
cw	circle of Willis
d	dentary bone
dc	deep cervical artery
dm	digastric muscle
dp	dorsal palatine artery
dp4	fourth deciduous premolar
ec	external carotid artery
ect	ectotympanic bone
fa	facial artery
fv	facial vein
gd	glandular duct
gV	trigeminal nerve ganglion
gX	vagus nerve ganglion
hc	hyoid cartilage
hf	hair follicle
hl	head length
i2	second lower incisor
i	incus
ic	internal carotid artery
is	inferior stapedial artery
jv	jugular vein
la	lingual artery
lac	lacrimal artery
lc	longis capitis muscle
lpt	lateral pterygoid muscle
m2	second lower molar
m3	third lower molar
m	malleus
ma c	mandibular canal
ma f	mandibular foramen
mc	Meckel’s cartilage
mtc	metaconid
mn	mandibular nerve
mo	molar
mpt	medial pterygoid muscle
mrm	medial rectus muscle
ms	masseter muscle
oa	occipital artery
op	occipital plexus
p3	third lower premolar
p4	fourth lower premolar
pa	posterior auricular artery
pag	parotid gland
par	parotid artery
pg	pituitary gland
pm	posterior meningeal artery
prs	proximal stapedial artery
ps	posterior stapedial artery
rc	Reichert’s cartilage
rcm	rectus capitis muscle
ri	ramus infraorbitalis artery
rl	recurrent laryngeal artery
rm	ramus mandibularis artery
rt	ramus temporalis artery
s	stapes
sa	superior alveolar artery
sc	superior constrictor muscle
scg	superior cervical ganglion
sm	stapedius muscle
smg	submandibular gland
ss	superior stapedial artery
ssm	superior stapedial meningeal artery
st	superficial temporal artery
stg	styloglossus muscle
sth	superior thyroid artery
stm	sterno(cleido)mastoid muscle
t	temporalis muscle
tb	talonid basin
tc	thyroid cartilage
tf	transverse facial artery
tg	thyroid gland
tt	tensor tympani muscle
tvp	tensor veli palatini muscle
va	vertebral artery
v	cranial nerve V or trigeminal
v2	second (maxillary) division of trigeminal nerve
v3	third (mandibular) division of trigeminal nerve
vii	cranial nerve VII or facial
viii	cranial nerve VIII or vestibulocochlear
x	cranial nerve X or vagus
xii	cranial nerve XII or hypoglossal

In order to digitally reconstruct the lower jaw from histological sections, slices between the anterior-most extent of the lower jaw and the posterior-most extent of the petrosal were sampled from the three coronally sectioned specimens (UMZC 2016.Histo.Po1, UMZC 2016.Histo.Po3, UMZC 2016.Histo.Po4; see Table 1). For these specimens, we used every third slice (i.e., approximately every 30 μa in Po1 and Po4 and every 36 μa in Po3) to create 3D models given software and memory limitations. The one sagittally sectioned specimen (UMZC 2016.Histo.Po2) was the smallest (Head Length [HL] 11 mm); therefore, we did not need to skip slices and could use the entire left side. While soft tissue structures such as blood vessels, nerves, and muscles can be confidently reconstructed using histologically prepared specimens, there is always the possibility of some distortion and missing sections when they are used to reconstruct anatomy in 3D. Therefore, we present our 3D figures as, at best, approximations of embryonic anatomy, sufficient for understanding basic anatomy, but not for precise measurements of anatomical structures within or between specimens. We discuss these specimens in order of size, from the smallest to the largest using the original histology numbers (Po2, Po1, Po4, Po3) written on each slide, so as to make it easier for future researchers to verify our observations. Slices with tissue damage were replaced with the immediately adjacent slice of highest quality. In the descriptions that follow, we frequently refer to the slice numbers where relevant anatomical structures are visible, using the format specimen ID, slide number, slice number. For example, “Po2 1-2” refers to UMZC specimen *Potamogale* 2016.Histo.Po2, the first slide in that series, and the second slice away from the slide label (“1-2”). In cases where there are more than two columns and rows, we start from the lower left of each slide, with the slide label situated at bottom. For example, “Po4 25-1-3” refers to the third slice up in the first column of slide #25 of specimen *Potamogale* UMZC.2016.Histo.Po4.

We used SPIERS Align 64-Bit (Sutton et al., 2012) to align the images for stack creation and for reconstructing the soft tissue anatomy. We used structures such as the nasal septal cartilage or the ventral edge of the dentary as markers to orient our alignment. For 3D reconstructions of jaws from histological slices, images were then cropped to one side of the animal and converted to grayscale in JPG format to minimize file size, and the resulting image sequence imported into Image-J. In the case of the sagittally sectioned Po2, we cropped regions caudal to the occiput. Stacks were then imported into Mimics to manually segment anatomical structures in 3D, based on differences in grayscale values between tissues and through comparison with the original colored stacks. We follow MacPhee (1981), Asher (2001) and Wible (2009) for anatomical terminology.

## Results

### *Potamogale* Po2 (HL = 11 mm)

**Figure 2:**
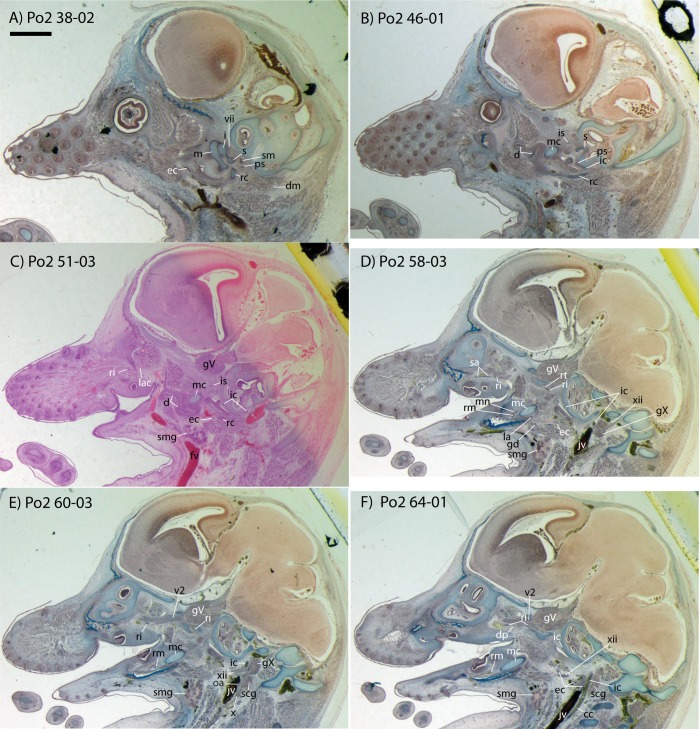
Sagittal sections of *Potamogale velox* (UMZC 2016.Histo.Po2) illustrating anatomical features discussed in the text for six sections, (A–F) in anatomical order from lateral (A) to medial (F), with each slide number and slice as indicated (e.g., “38-02” means slide #38, slice #2). For abbreviations see Table 3. Scale bar at top left represents approximately 1 mm.

**Figure 3:**
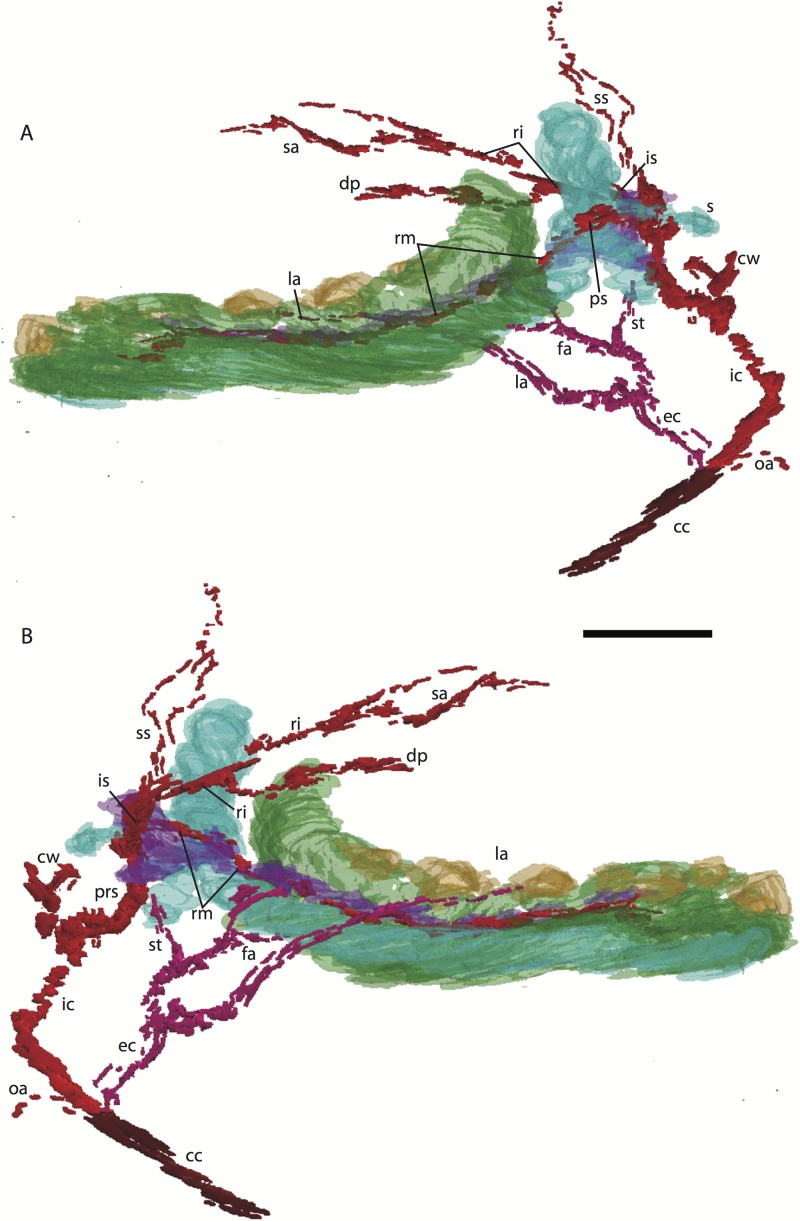
3D reconstruction of the lower jaw and common, internal, and external carotid arteries of *Potamogale velox* (UMZC 2016.Histo.Po2) in dorsolateral (A) and ventromedial (B) views. Common carotid artery = dark red, external carotid branches (including occipital) = pink, internal carotid branches = bright red, dentary = green, teeth = orange, Meckel’s cartilage (with attached ear ossicles) = blue, mandibular nerve = purple; scale bar is approximately 1 mm.

This specimen is the smallest, least ossified, and therefore presumably youngest in our developmental series (Table 1). Ossifications are evident in the anterior part of the braincase, and in the palate, maxilla, premaxilla, and some of the squamosal. The ear ossicles are cartilaginous and the malleus is broadly continuous with Meckel’s cartilage, which is relatively large compared with the dentary and extends throughout the length of the jaw. Interestingly, to the extent that this specimen can be said to have a jaw joint, it would be between incus and malleus, not squamosal and dentary (Po2 38-1; see Fig. 2A and Sánchez-Villagra et al., 2002). Ossification of the dentary is evident ventral and anterior to Meckel’s cartilage, and is more extensive anteriorly (extending dorsal to Meckel’s cartilage, close to the symphysis) than posteriorly. Ossification is more advanced in the coronoid process than in the mandibular condyle (Po2 46-3; Fig. 2B). Nine tooth buds of varying sizes are evident in the lower jaw (two of which are appear very close to each other in the first and penultimate tooth reconstruction in Fig. 3), all connected by a continuous dental lamina (Fig. 3).

### Common and external carotids

Bifurcation of the common carotids occurs anterior to the jugular ganglion of the vagus nerve, ventral to the superior cervical ganglion, and posterior to the jugular vein (Po2 63-3; Fig. 2F). The external carotid gives off an occipital branch immediately distal to the bifurcation with the internal carotid. The occipital courses between the jugular vein (anteriorly) and vagus nerve (posteriorly) while descending slightly in the neck (Po2 60-3; Fig. 2E), and begins to ramify and become indistinct lateral and inferior to the jugular vein. After giving off the occipital artery, the external carotid courses anteriorly, giving rise to a lingual branch dorsal to the hypoglossal nerve (Po2 65-3) and lateral to the cartilaginous precursor of the hyoid bone. This lingual branch then proceeds to the posterior margin of the tongue, traversing ventral to the greater cornu of the hyoid bone (Po2 72-2) and continuing anteriorly to the hyoid to enter the hyoglossus muscle at its posterior margin (Po2 78-2) and also supplying styloglossal and genioglossal musculature. After giving off the lingual artery, the external carotid courses laterally, continuing for some of its length dorsal to the digastric muscle and anterior to Reichert’s cartilage (Po2 50-3). After passing ventral to a diverticulum of the middle ear space, it supplies a trunk for the facial, transverse facial, and superficial temporal branches (Po2 38-2; Fig. 2A).

### Internal carotid and stapedial

Having diverged from the external carotid, the internal carotid travels dorso-laterally, entering the middle ear dorsal to the digastric and anterior to the jugular vein (Po2 52-1; Fig. 2C). The internal carotid then travels anteromedially along the ventral surface (or ventrum) of the pars cochlearis and gives off the proximal stapedial artery (Po2 46-1; Fig. 2B). At the antero-medial edge of the ventral pars cochlearis, the internal carotid enters the braincase via a cartilaginous carotid foramen (Po2 74-1) between the cartilaginous basisphenoid and petrosal. The internal carotid pierces the dura mater and supplies the Circle of Willis just medial to the trigeminal ganglion and anterior to the pituitary (Po2 79-1).

The proximal stapedial artery gives off a posterior branch (Po2 42-6) immediately prior to passing through the obturator foramen of the stapes (Po2 43-6). The posterior stapedial artery courses posterolaterally, supplying the stapedius muscle, and then ramifies to supply structures posterior, lateral, and ventral to the cartilaginous pars canalicularis of the petrosal (Po2 29-2). This may include a posterior auricular branch but the vessels are too indistinct to be certain.

Upon passing through the obturator foramen of the stapes, a small superior stapedial branch is evident which courses into the braincase. It is slightly more evident on the left side (Po2 137-4) than right (Po2 41-1). The inferior stapedial bifurcates into rami infraorbitalis and mandibularis (Po2 54-3) immediately posterior and ventral to the mandibular division of the trigeminal nerve (CN-V3).

The ramus infraorbitalis (or infraorbital artery) is the continuation of the inferior stapedial, distal to its bifurcation with the ramus mandibularis (i.e., inferior alveolar or mandibular artery). As the ramus infraorbitalis passes medial to the mandibular nerve, it gives off a small temporal branch that courses dorsal to fibers of the internal pterygoid muscle and ramifies towards fibers of the temporalis muscle (Po2 58-1; Fig. 2D). Distal to its origin from the inferior stapedial, the ramus infraorbitalis enters the cavum epiptericum via a large foramen in the cartilaginous alisphenoid bone and courses anteriorly ventral to the trigeminal ganglion (Po2 60-3; Fig. 2E). As the trigeminal gives off the large maxillary nerve (CN-V2), the ramus infraorbitalis leaves the cavum epiptericum with V2 dorsal to it, via the sphenorbital fissure, and supplies the dorsal palatine artery (Po2 64-3; Fig. 2F). No sign of a sphenopalatine artery supplying the posterior nasal fossa is evident in this specimen.

Distal to the dorsal palatine, the ramus infraorbitalis supplies a trunk for the superior alveolar artery and distal branches beyond the infraorbital canal. Before it reaches the infraorbital foramen, the ramus infraorbitalis artery supplies a large superior alveolar branch (Po2 58-3; Fig. 2D), which courses dorsal to the dental lamina, giving off branches to it. The ramus infraorbitalis continues anteriorly with fibers of the maxillary nerve and gives off a small but distinct lacrimal branch (Po2 53-3) which moves anterodorsally towards the optic neurovascular bundle and eye musculature, ramifying just anteromedial to the eye itself (Po2 51-3; Fig. 2C). There is no sign of lacrimal glandular tissue in Po2. The ramus infraorbitalis continues anteriorly through the ossified infraorbital foramen in the maxilla, providing large vascular rami to supply the maxillary part of the face and its densely packed, sensory hair follicles (Po2 48-1).

### Mandibular nerve and artery

The mandibular nerve comprises the third division of the trigeminal nerve (CN-V3). In Po2 it leaves the braincase via the cartilaginous foramen ovale (Po2 56-1), anterior to the petrosal and ventrolateral to the exit point of CN-V2 via the sphenorbital fissure. It then courses towards the dentary, passing ventral to a slip of the lateral pterygoid muscle and dorsal to Meckel’s cartilage (Po2 56-3), accompanied by the ramus mandibularis (i.e., inferior alveolar or mandibular artery). A distinct foramen in the ossifying dentary for nerve and vessel is not yet obvious, but both structures are coursing within the dentary, anterior to Meckel’s cartilage, by slice Po2 59-3 (Fig. 2D).

### *Potamogale* Po1 (HL = 13 mm)

**Figure 4:**
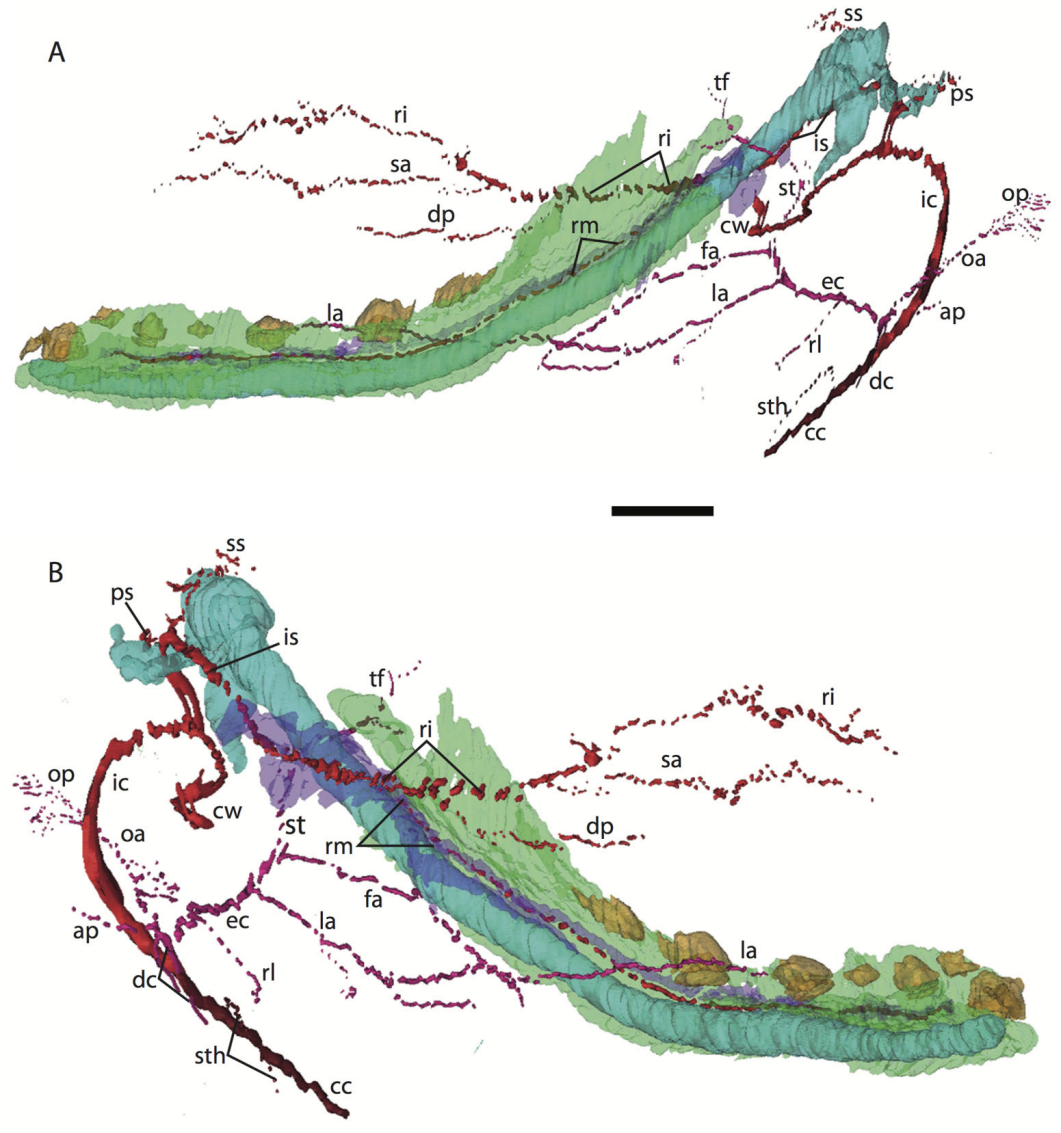
3D reconstruction of the lower jaw of *Potamogale velox* (UMZC 2016.Histo.Po1) in posterolateral (A) and anteromedial (B) views. Colors are as in Fig. 3; scale bar is approximately 1 mm.

The dentary, maxilla, premaxilla and palatine of Po1 are more completely ossified than in Po2, and the squamosal, pterygoid alae of the sphenoid, jugal, and frontal bones are beginning to ossify; other skull bones are largely cartilaginous. Meckel’s cartilage is large and conspicuous, and the cartilaginous malleus remains attached to its posterior end. In comparison to Po2 (HL 11 mm), Meckel’s cartilage is slightly smaller relative to the dentary, and follows a relatively straight path from anterior to posterior, without a medial deflection around the mandibular condyle at its posterior margin. A dental lamina connecting eight tooth buds, with an additional, unconnected, tooth-bud like invagination apparent at the anterior margin of the dentary (Po1 13-02), is visible in each dentary (Fig. 4).

### Common and external carotids

**Figure 5:**
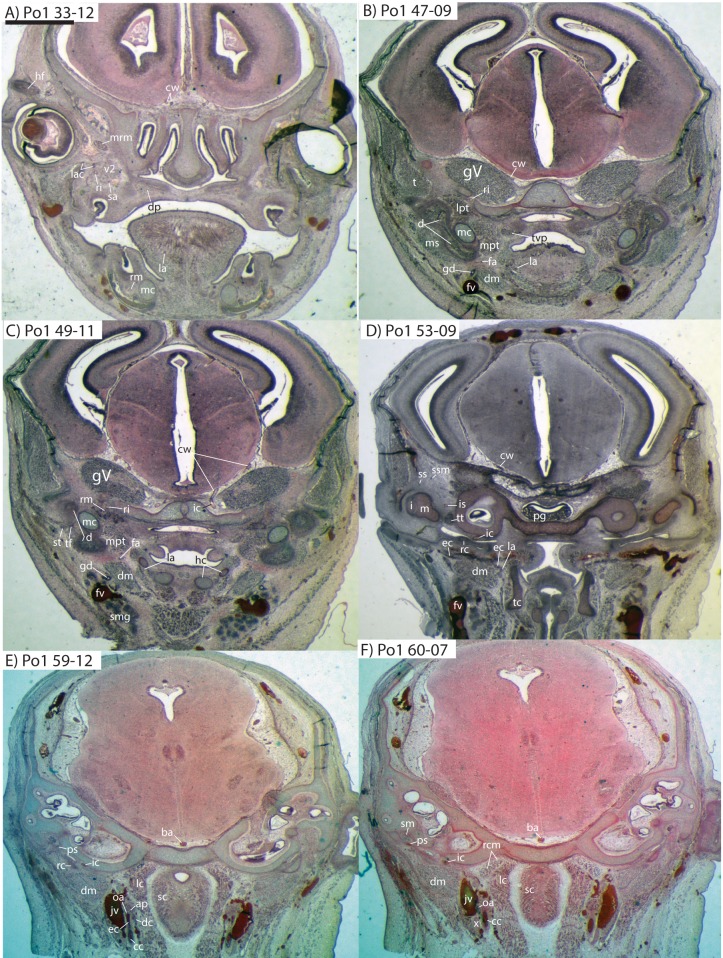
Coronal sections of *Potamogale velox* (UMZC 2016.Histo.Po1) illustrating anatomical features discussed in the text for six sections. (A–F) with each slide number and slice as indicated (e.g., “33-12” means slide #33, slice #12). For abbreviations see Table 3. Scale bar at top left represents approximately 1 mm.

The common carotid artery bifurcates into the external and internal carotids lateral to the longis capitis muscle and medial to the jugular vein (Po1 59-12; Fig. 5E). Proximally (and ventrally) to the carotid bifurcation is the origin of the superior thyroid artery (Po1 58-08; Fig. 4), which uniquely among our histological sample has an independent origin from the common carotid (as opposed to the external carotid in specimens Po3 and Po4). The external carotid artery then supplies a common trunk for the deep cervical, ascending pharyngeal, and occipital arteries (Po1 59-12; Fig. 5E). The deep cervical courses ventromedially and ascending pharyngeal courses dorsomedially, both lateral to longis capitis muscle and anterior to the internal carotid. The occipital courses dorsomedially and caudally, moving posteriorly to the jugular vein (Po1 60-07; Fig. 5F). As it continues laterally, it supplies branches towards the sternomastoid muscle and then ramifies into a vascular plexus occupying a fascial plane medial to muscular fibers of sternomastoid, ventral to the digastric, and medial to the superior oblique (Po1 66-07).

The external carotid leaves its junction with the internal carotid to course anterodorsally. The next branch to leave the external carotid, distal to the trunk for the deep cervical, ascending pharyngeal, and occipital arteries, is a small branch for the recurrent laryngeal (Fig. 4). This artery courses ventrally to enter the thyroid area via a foramen in the thyroid cartilage (Po1 55-0). At a point lateral to the dorsal margin of the thyroid cartilage and medial to the digastric, the external carotid gives off the lingual artery (Po1 53-09; Fig. 5D). The lingual artery courses ventrolateral to the cartilaginous hyoid bone and enters the body of the tongue anterior to the hyoid (Po1 47-09; Fig. 5C).

Following its divergence, the external carotid trunk for the facial and temporal arteries travels dorsolaterally, dorsal to the digastric (Po1 53-06; Fig. 5C). It then passes posterior to the dentary, between the jaw angle (inferiorly) and mandibular condyle (superiorly), and divides into temporal branches coursing superiorly and a facial branch coursing anteromedially (Po1 49-08; Fig. 5C). This transverse facial branch travels initially among fibers of the masseter muscle, along the lateral margin of the coronoid process of the dentary (Po1 45-07). A large duct connecting the submaxillary and sublingual glands is conspicuous (Po1 47-09; Fig. 5B) dorsal to the facial vein, coursing anteromedially towards the sublingual gland between the digastric muscle (ventrally) and partly-ossified dentary (dorsally).

### Internal carotid and stapedial

The internal carotid courses dorsolaterally from the carotid bifurcation, towards the ventrum of the pars cochlearis of the petrosal (Po1 61-02). As it approaches the oval window and the cartilaginous stapes, the internal carotid bifurcates into the proximal stapedial and internal carotid proper (Po1 57-10). The former is a large artery that supplies a small posterior stapedial ramus just before passing through the obturator foramen of the stapes (Po1 58-05). The latter is also a large-caliber artery that courses dorsomedially towards the carotid foramen, passes through it just anteromedial to the petrosal (Po1 51-10), and pierces the dura mater to supply the Circle of Willis (Po1 48-13; Fig. 5C).

After its origin from the proximal stapedial artery immediately ventral to the stapes, the posterior stapedial courses posterolaterally, posterior to fibers of the facial nerve, ventral to the origin of the stapedius muscle (Po1 60-04; Fig. 5F), and bifurcates into two branches: one that travels anteriorly towards the pinna (posterior auricular) and another that travels posterodorsally ramifying in the superficial occipital region. Po1 shows a small but clear superior stapedial branch, which bifurcates from the larger inferior stapedial artery ventral to the facial nerve, medial to the cartilaginous incus, and dorsal to the tensor tympani muscle (Po1 55-11). The superior stapedial divides further into two small branches dorsal to the incus-malleus articulation and largely membranous cavum epiptericum (Po1 53-12; Fig. 5D): one leading dorsomedially towards the meninges of the braincase, and the other dorsolaterally towards posterior fibers of the temporalis muscle.

After its bifurcation with the superior stapedial, the inferior stapedial artery courses antero-medially, lateral to the pars cochlearis and fibers of the tensor tympani muscle and medial to the proximal aspect of the malleus as it grades into Meckel’s cartilage (Po1 52-08; Fig. 5D). It bifurcates into rami mandibularis and infraorbitalis just posterior to fibers of the mandibular nerve (CN V3) and ventral to the foramen ovale (Po1 49-14; Fig. 5C). Shortly after this bifurcation and dorsal to fibers of the lateral pterygoid muscle, the ramus infraorbitalis gives off a small middle meningeal branch (Po1 48-04) that enters the braincase via the foramen ovale. The ramus infraorbitalis then re-enters the braincase via the cartilaginous alisphenoid canal, ventral to the body of the trigeminal nerve ganglion (Po1 47-03; Fig. 5B). The artery then travels anteriorly, ventral to fibers of the maxillary nerve (CN V2), and leaves the braincase via the unossified sphenorbital fissure (Fig. 5A). As it does, a buccal branch leaves it laterally, posterior to the dentary, and a dorsal palatine artery leaves it medially (Po1 39-07), coursing towards the descending palatine canal. As reported in other specimens of *Potamogale* (Asher, 2001), the dorsal palatine does not appear to supply a major sphenopalatine branch in Po1, despite its close proximity to the posteroventral nasal fossa. The dorsal palatine continues anteriorly to supply the greater palatine artery in the roof of the mouth.

Distal to the dorsal palatine and buccal arteries, the ramus infraorbitalis continues anteriorly with the infraorbital nerve, and gives off a superior alveolar artery (Po1 35-08; Fig. 5A) followed by a lacrimal branch which courses towards the eye (Po1 33-06; Fig. 5A), with no sign of any lacrimal glandular tissue. At this point the ramus infraorbitalis has entered the infraorbital canal, medial to the partly ossified maxilla and lateral to the cartilaginous nasal capsule. Inside the canal, the ramus infraorbitalis ramifies along with fibers of the infraorbital nerve and leaves it (Po1 30-06) to supply the large number of hair follicles and facial muscles, densely packed into the anterior rostrum.

### Mandibular artery and nerve

As previously noted, the inferior stapedial artery bifurcates into ramus infraorbitalis and mandibularis ventral to the cartilaginous foramen ovale, amidst fibers of CN-V3 (Po1 49-11; Fig. 5C). The ramus mandibularis courses ventrolaterally, at first dorsal to Meckel’s cartilage and ventral to the lateral pterygoid muscle, gradually moving lateral to Meckel’s cartilage as the artery enters the body of the dentary via an as-yet unossified mandibular foramen (Po1 44-03). The ramus mandibularis continues anteriorly, lateral to Meckel’s cartilage and ventral to the dental lamina, providing small branches to supply these and other nearby structures throughout the length of the partly-ossified dentary.

### *Potamogale* Po4 (HL = 16 mm)

**Figure 6:**
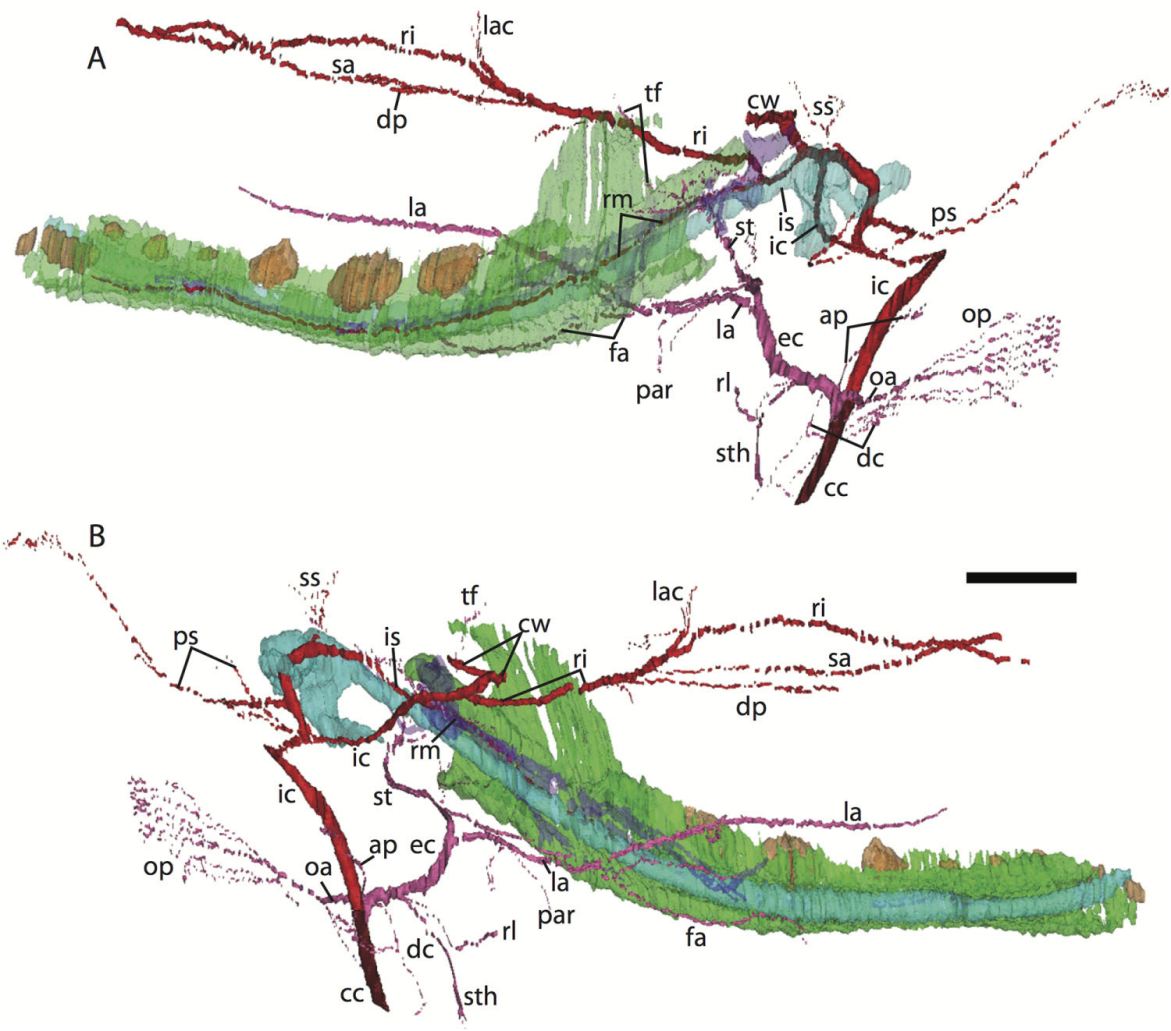
3D reconstruction of the lower jaw of *Potamogale velox* (UMZC 2016.Histo.Po4) in lateral (A) and medial (B) views. Colors are as in Fig. 3; scale bar is approximately 1 mm.

This specimen shows more ossification around the periphery of endochondral cranial elements than Po1, including the pterygoid alae, basioccipital, basisphenoid, alisphenoid, vomer, and palatines (but not ethmoid or petrosals). Dermal elements of the braincase and the dentary are substantially ossified, except for the mandibular condyles. Meckel’s cartilage extends from its solid connection to the malleus through the length of the dentary, but comprises a smaller proportion of the lower jaw compared to that of Po1 (Fig. 6). As in Po1, eight tooth buds are clearly apparent on each side of the dentary, with a diminutive, tooth-bud like invagination at its anterior margin (Po4 4-1-5). The connections via the dental lamina between each tooth bud are now weaker than those present in Po1 and Po2.

### Common and external carotids

**Figure 7:**
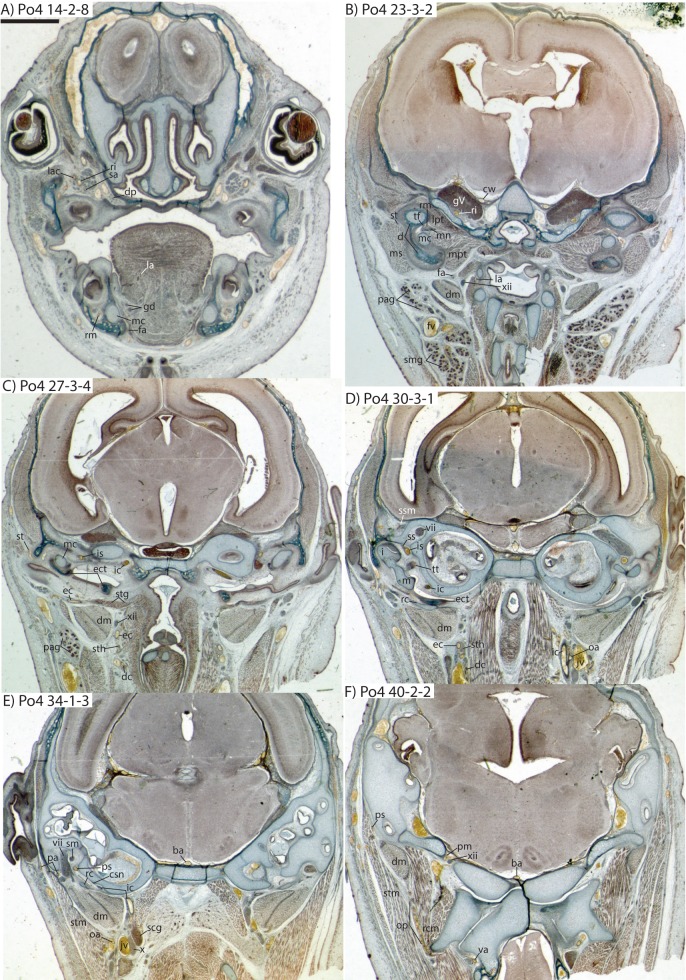
Coronal sections of *Potamogale velox* (UMZC 2016.Histo.Po4) illustrating anatomical features discussed in the text for six sections, (A–F) with each slide number and slice as indicated (e.g., “14-2-8” means slide #14, row #2, column #8). For abbreviations see Table 3. Scale bar at top left represents approximately 1 mm.

**Figure 8:**
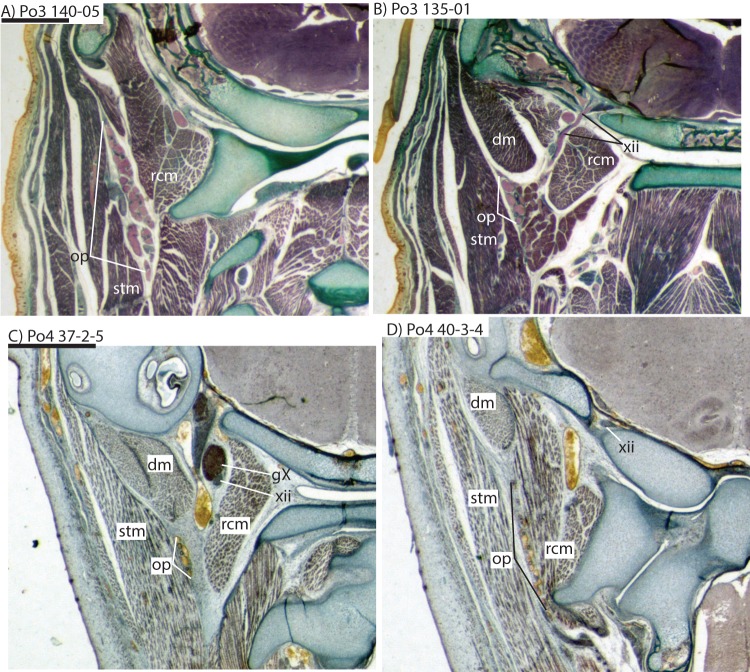
Coronal sections illustrating vascular retes derived from the occipital artery in *Potamogale velox* (Po3: (A, B) and Po4: (C, D)), with each slide number and slice as in Figs. 2, 5 and 7. Figure 10F exhibits similar vascular structures derived from the deep cervical and ascending pharyngeal arteries in Po3. For abbreviations see Table 3. Scale bars at left represent approximately 1 mm.

The carotid bifurcates into internal and external branches lateral to the longis capitis muscle and medial to the jugular vein (Po4 32-1-6). The occipital artery branches from the external carotid very close to the carotid bifurcation (Po4 32-2-2; Fig. 7D), and courses posterolaterally towards the medial margin of the sternomastoid muscle and ventral to the digastric. It gives off a number of small branches that ascend towards the former and descend towards the latter before ramifying into a plexus of arteries and veins that are most dense in a fascial plane ventral to the digastric, lateral to rectus capitis, and medial to sternomastoid muscles (Po4 40-2-2; Fig. 7F). In this vascular plexus, each arterial branch is surrounded by larger, thin-walled veins (Fig. 8). The plexus terminates as capillaries lateral and posterior to the alae of the atlas, and do not distally rejoin each other as a central vessel.

Just distal to the origin of the occipital artery, and still close to the carotid bifurcation, the external carotid gives off an ascending pharyngeal branch (Po4 31-3-4) that courses dorsomedially towards the cranial base, and a deep cervical artery that courses ventrally, bifurcates, and eventually ramifies into a vascular rete anteromedial to the carotid bifurcation (Po4 32-1-6). The superior thyroid and recurrent laryngeal arteries have origins very close to each other from the external carotid, just distal to the origin of the deep cervical artery (Po4 30-3-1; Fig. 7D). Both course ventromedially; the superior thyroid is larger than recurrent laryngeal and courses below the latter, towards the thyroid gland (Po4 26-3-1). The recurrent laryngeal shows a medially directed curve, dorsal to the superior thyroid, and eventually pierces the thyroid cartilage (Po4 25-1-6).

The external carotid courses anterodorsally until the posterodorsal margin of the cartilaginous hyoid (Po4 25-3-7), at which point it gives off the lingual artery, which follows the dorsum of the hyoid towards the posterior tongue (Po4 21-3-2; Fig. 7B). After giving off the lingual artery, the external carotid turns laterally and courses dorsal to the digastric and ventral to the ossifying ectotympanic bone (Fig. 7C), and bifurcates into a small, anteriorly running facial artery and a posteriorly running trunk for superficial temporal and transverse facial arteries (Po4 27-2-5). The superficial temporal artery courses posterodorsally and gives off branches that run towards the mandibular condyle and the tissues posterior to the dentary (Po4 26-1-3); the latter comes very close to the ramus mandibularis (supplied by the inferior stapedial), dorsal to Meckel’s cartilage (Po4 24-1-3; Fig. 7B) and may anastomose with it at a capillary level (although major connections between the arteries are not evident here). The main trunk of the superficial temporal artery continues posterodorsally and ramifies lateral to the squamosal articulation for the jaw (Po4 25-1-3), sending branches anteriorly into fibers of the temporalis muscle, lateral to the posterior aspect of the coronoid process.

The facial artery leaves the external carotid dorsal to the digastric, medial to the styloglossus. Shortly after its origin, the facial artery gives off a laterally coursing branch that supplies the submandibular and parotid glands (Po4 23-2-1; Fig. 7B). The facial then continues anteriorly along the inferior margin of the dentary, ventral to the medial pterygoid muscle and dorsal to the digastric (Po4 23-3-5), gradually reaching the lateral aspect of the mylohyoid muscle as it travels along the length of the jaw (Fig. 7A), extending ventral to the sublingual gland and ramifying into it (Po4 13-3-5).

### Internal carotid and stapedial

 The internal carotid initially courses posterodorsally from the carotid bifurcation. As it approaches the cartilaginous petrosal, it gives off a small, posterior meningeal branch (Po4 34-1-3; Fig. 7F) that travels briefly dorsomedial to the vagus ganglion, and courses dorsomedially adjacent to the hypoglossal nerve to enter the braincase via the hypoglossal foramen in the cartilaginous occipital (Po4 39-3-4; Fig. 7F). The internal carotid then begins to course anterolaterally along the ventrum of the cartilaginous pars cochlearis of the petrosal, ventromedial to a small nerve bundle from the superior cervical ganglion (Po4 33-3-4; Fig. 7E). It then bifurcates into the proximal stapedial and internal carotid proper; the former gives rise to a large posterior stapedial artery before traversing the obturator foramen of the cartilaginous stapes (Po4 32-3-4). The posterior stapedial artery courses laterally out of the middle ear space, posterior to the motor root of the facial nerve (Po4 34-1-6; Fig. 7E), and divides into a conspicuous posterior auricular artery that courses anteriorly towards the pinna, and another branch coursing posterodorsally around the mastoid region, dorsal to the origin of the digastric, medial to the origin of sternomastoid musculature (Po4 41-3-7; Fig. 7F).

After passing through the obturator foramen of the stapes, the proximal stapedial artery passes ventral to the facial nerve and then sends off a small but conspicuous superior stapedial ramus (Po4 30-3-1; Fig. 7D). The superior stapedial enters the braincase through a foramen in the ossified squamosal portion of the epitympanic recess; this in turn divides into a meningeal branch that courses dorsomedially towards the brain (Po4 30-3-1; Fig. 7D), and a temporal branch that re-exits the braincase via a space between ossifications of the squamosal and parietal (Po4 28-3-1). As observed in other specimens of *Potamogale* (Asher, 2001) branches of the superior stapedial ramus are small and limited to supplying tissues in and near the epitympanic recess.

The inferior stapedial is a major artery that courses anteromedially along the roof of the middle ear, medial to the cartilaginous malleus-Meckel’s cartilage, leaving the middle ear dorsal to the ossified ectotympanic bone and ventral to the partially ossified alisphenoid (Po4 26-2-5). Ventral to the foramen ovale, and amidst fibers of the mandibular division of the trigeminal nerve (CN V3), the inferior stapedial bifurcates into rami mandibularis and infraorbitalis (Po4 26-1-3). The ramus infraorbitalis then courses dorsomedially to enter the cavum epiptericum, ventral to the trigeminal nerve, via the alisphenoid canal (Po4 24-3-1). Immediately before entering the canal, the ramus infraorbitalis sends a small branch dorsolaterally, dorsal to fibers of the lateral pterygoid muscle, towards temporalis musculature anterior to the mandibular condyle and posterior to the coronoid process (Po4 25-1-3). The ramus infraorbitalis then courses within the cavum epiptericum, ventral to the maxillary and ophthalmic divisions of the trigeminal (Fig. 7B). It leaves the largely cartilaginous sphenorbital fissure dorsal to the origin of the medial pterygoid and lateral to the ossified pterygoid ala of the sphenoid bone (Po4 19-3-8). It then sends a small buccal branch laterally and a dorsal palatine branch medially (Po4 18-1-2). The latter courses inferomedially towards the posterior palate via the palatine canal (Fig. 7A), but does not appear to send any major sphenopalatine branches into the nasal fossa. Just prior to entering the partly-ossified infraorbital canal, the ramus infraorbitalis sends off a branch that ramifies towards the orbital and lacrimal regions (Po4 14-1-5; Fig. 7A), despite (as in other specimens) no obvious signs of lacrimal glandular tissues. The superior alveolar artery arises from the ramus infraorbitalis as the latter enters the infraorbital canal (Po4 14-2-8; Fig. 7A), and continues anteroventrally to reach the upper dentition through the maxilla. By the time the ramus infraorbitalis reaches the anterior exit of the infraorbital canal, it has divided into at least four arterial branches (Po4 11-4-1) that continue forward to supply the tissues of the face and rostrum.

After giving off the proximal stapedial artery, the internal carotid proper courses anteromedially along the ventrum of the cartilaginous pars cochlearis of the petrosal. Without giving off any major branches, it reaches the carotid foramen, between a partially ossified basisphenoid medially and the cartilaginous anterior pole of the cochlea laterally (Po4 27-3-4; Fig. 7C). It proceeds dorsomedially into the braincase, piercing the dura mater to join the Circle of Willis (Po4 25-1-3; Fig. 7B). Posterior supply to the Circle of Willis is also evident from the basilar artery in the midline of the floor of the posterior braincase (Fig. 7F), which is in turn supplied by the two vertebral arteries. These travel through the vertebral foramina of the cervical vertebrae, including the atlas (Po4 43-4-4; Fig. 7F), and enter the braincase via the foramen magnum (Po4 42-3-5).

### Mandibular artery and nerve

Following the bifurcation of inferior stapedial into rami mandibularis and infraorbitalis, the ramus mandibularis courses ventrolaterally, just behind fibers of the mandibular division of the trigeminal nerve, towards the dentary. It enters the body of the dentary via an as-yet incompletely ossified mandibular foramen, dorsal to Meckel’s cartilage (Po4 23-3-2; Fig. 7B). The mandibular canal is ossified dorsally, ventrally, and laterally for most of its length. The artery is bounded dorsally by CN V3 and medially by Meckel’s cartilage, the largest remaining part of the jaw that is unossified (Po4 17-3-7). Areas of the dentary dorsal to the developing tooth buds (and alveoli) remain unossified, as are mental foramina towards the anterolateral aspect of the jaw. Otherwise, no major canals or foramina are evident except for the mandibular canal and alveoli.

### *Potamogale* Po3 (HL = 24 mm)

**Figure 9:**
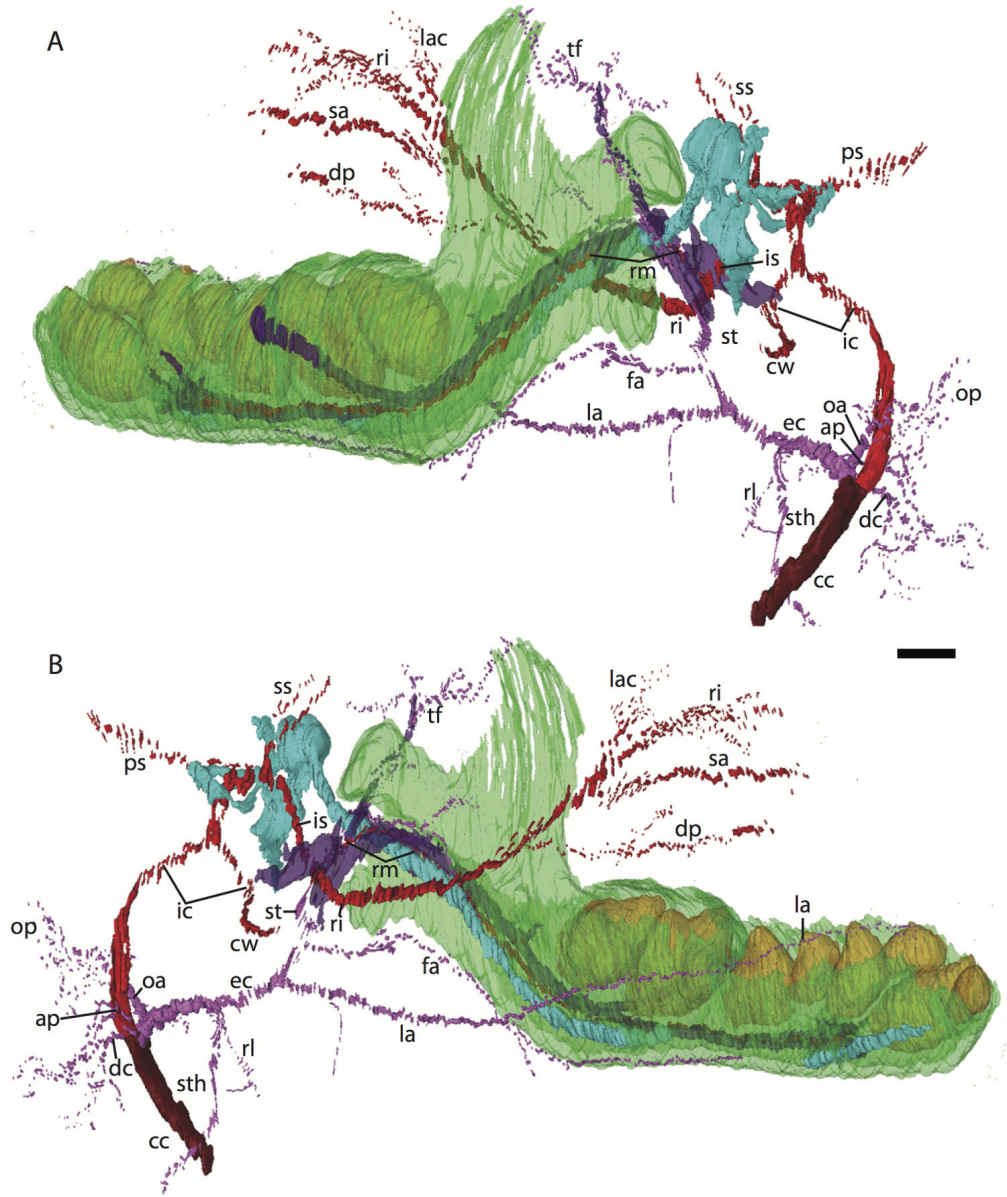
3D reconstruction of the lower jaw of *Potamogale velox* (UMZC 2016.Histo.Po3) in posterolateral (above) and anteromedial (below) views. Colors are as in Fig. 3; scale bar is approximately 1 mm.

Most of the bones of the skull have ossified or are in the process of ossifying; only parts of the basisphenoid, occipital, petrosal, ethmoid, and mandibular condyles remain largely unossified. Meckel’s cartilage retains a narrow connection to the malleus, and due to the relatively large and mediolaterally expanded mandibular condyle, is medially deflected around the condyle as it continues anteriorly towards the dentary. Eight well developed tooth buds are apparent in their crypts, some close to eruption. Based on the eruption sequence known for *Potamogale* (Asher & Lehmann, 2008; Asher & Olbricht, 2009), we regard these as deciduous incisors 1-3 (di1-3), the deciduous canine (dc), deciduous premolars 2-4 (dp2-4), and the first molar (m1). As in the smaller specimens described above, there is a minute, ectodermal, tooth-bud like invagination just dorsal to the di1 toothbud (Po3 24-4). In contrast to the other tooth loci, this structure exhibits no signs of enamel or dentin formation, and is presumably resorbed in larger, more developed specimens. As Meckel’s cartilage reaches the ventromedial aspect of the dentary, it becomes surrounded by ossifications of the dentary; at a point ventral to the toothbud of dp4, it is completely resorbed (Po3 65-4). Farther anteriorly, medial to the developing dc and dp2 tooth buds, it is again apparent (Po3 44-4) and continues on each side towards the mandibular symphysis. Crowns of the di3 and dc are the closest to eruption (Fig. 9).

### Common and external carotids

**Figure 10:**
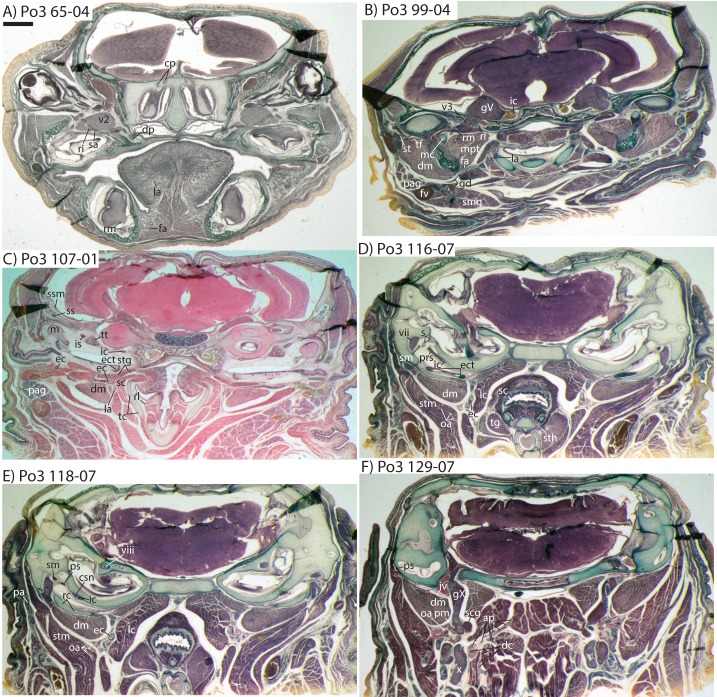
Coronal sections of *Potamogale velox* (UMZC 2016.Histo.Po3) illustrating anatomical features discussed in the text for six sections, (A–F) with each slide number and slice as indicated (e.g., “65-04” means slide #65, slice #4). For abbreviations see Table 3. Scale bar at top left represents approximately 1 mm.

The common carotid divides into internal and external carotids medial to the jugular vein and lateral to the longus capitis muscle (Po3 123-08). The occipital artery also arises here, slightly closer to the lumen of what will form the external carotid. The occipital artery courses posterolaterally from its origin, behind the jugular vein and towards the sternomastoid muscle. It then divides into anteriorly and posteriorly directed branches; the former ramifies into a plexus, initially between the sternomastoid (ventrally), jugular vein (medially), and digastric (dorsally, see Po3 122-03 and Fig. 10E). The anterior occipital branch then ramifies and several of its tributaries are evident entering the body of the sternomastoid muscle and course anteroventrally within its muscular fibers (Po3 114-12; Fig. 10D). The posterior occipital branch is evident medial to the vagus nerve and superior cervical ganglion. As it approaches the latter, it sends off small posterior meningeal branches that ramify and follow the hypoglossal nerve towards the hypoglossal foramen (Po3 134-10). As observed in Po1 and Po4, the occipital artery contributes to a vascular plexus, again sandwiched between fibers of the digastric (dorsally) and sternomastoid (ventrally). Farther posteriorly, the plexus approaches the lateral margin of the rectus capitis muscle (Po3 139-08; Fig. 8A). While it is also derived from the occipital trunk, this posterior occipital plexus ramifies separately from that mentioned above derived from branches of the anterior occipital artery. The posterior occipital plexus further differs from its anterior counterpart in remaining primarily within a facial plane between muscles (Fig. 8), although some branches do penetrate intra-muscular spaces such as the sternomastoid.

Very close to its origin, the external carotid gives off two arteries medially, the deep cervical (coursing ventrally, Po3 131-04) and the ascending pharyngeal (coursing dorsally, Po3 129-07). Interestingly, and as observed for the occipital branches, these also appear to ramify and course alongside a net of veins (Fig. 10F), comprising a third vascular plexus in the neck region (Figs. 8, 9 and 10F).

The external carotid then courses anteriorly between the digastric (laterally) and superior constrictor (medially) muscles. A recurrent laryngeal branch that courses ventromedially, towards the larynx, is evident ventral to the external carotid in Po3 116-07 and ramifies to supply a small vessel that pierces the thyroid cartilage just ventral to the recurrent laryngeal branch of the vagus nerve (Po3 110-08; Fig. 10C). A larger, superior thyroid artery is evident branching off immediately distal to the recurrent laryngeal, where it courses ventromedially, between the superior constrictor medially and thyroid gland laterally (Po3 116-07; Fig. 10D). The next major branch of the external carotid is apparent as it approaches the dorsolateral margin of the thyroid cartilage, at which point it divides into the lingual artery and trunk for the transverse facial and superficial temporal arteries (Po3 107-06; Fig. 10C). The lingual artery follows the dorsum of the cartilaginous hyoid and passes anterior to it to enter the body of the tongue (Po3 91-01; Fig. 10B).

The trunk for the transverse facial, superficial temporal, and facial branches of the external carotid takes a sharp lateral turn distal to the origin of the lingual artery, ventral to the styloglossus muscle and dorsal to the digastric (Po3 107-01; Fig. 10C). This trunk courses ventral to the external. auditory meatus and gives off a small branch posterior to the dentary, which (as described above for Po4) then moves towards Meckel’s cartilage and approaches the ramus mandibularis (Po3 99-04; Fig. 10B), which as noted above is supplied by the inferior stapedial artery. The transverse facial artery divides from the superficial temporal ventrolateral to the mandibular condyle, dorsal to fibers of the masseter muscle and medial to the facial nerve (Po3 102-07). The transverse facial then courses anteriorly lateral to the coronoid process, whereas the superficial temporal courses anterodorsally amidst muscular fibers of the temporalis and towards the dorsum of the braincase.

The facial artery also arises from the distal branches of the external carotid, inbetween the styloglossus (dorsally) and digastric (ventrally; see Po3 107-01; Fig. 10C). After its origin, the facial courses anteriorly on the dorsum of the digastric, towards the ventrum of the dentary, branching en route to supply tissues of the submandibular gland (Po3 99-01; Fig. 10B). Ducts connecting the submandibular to the sublingual gland are similar in appearance to arterial lumina (e.g., Po3 96-10; Fig. 10B), but are distinct and discontinuous from them. The facial artery courses anteriorly, along the ventromedial aspect of the dentary, and extends anteriorly towards the sublingual gland, base of the tongue (e.g., Po3 69-01; Fig. 10A), and mandibular symphysis.

### Internal carotid and stapedial

After its bifurcation from the common carotid, the internal carotid courses dorsally. It follows a path just medial to the carotid sympathetic nerve along the ventrum of the pars cochlearis (Po3 120-09; Fig. 10E), continuing posterolaterally until it divides into the proximal stapedial and internal carotid proper, just ventromedial to the cartilaginous stapes, within the middle ear space (Po3 117-04; Fig. 10D). The proximal stapedial gives off a large posterior stapedial branch, which courses posterolaterally and gives rise to anterior and posterior branches. The anterior branch leaves the middle ear ventral to fibers of the stapedius muscle and dorsal to the facial nerve (Po3 120-11), and terminates via a posterior auricular artery which turns anterolaterally and ramifies towards the pinna (Po3 120-09; Fig. 10E). The posterior branch courses posteriorly, just ventral to the origin of the stapedius muscle and ramifies along the external surface of the mastoid region amidst fibers of the digastric and sternomastoid muscles (Po3 127-03).

Soon after coursing through the obturator foramen of the stapes, ventral to the facial canal and dorsolateral to the tensor tympani muscle, the proximal stapedial artery divides into small superior and large inferior stapedial rami (Po3 109-04; Fig. 10C). The superior stapedial leaves the middle ear space via a gap between the ossified squamosal (defining the roof of the epitympanic recess) and unossified petrosal (Po3 108-07). Once above the epitympanic recess, the superior stapedial gives off a small meningeal branch (Fig. 10C); meningeal and other superior stapedial branches course dorsally towards the interior margins of the braincase, but appear to terminate among the meninges and diploë without providing major blood supply to more anterior cranial structures.

The inferior stapedial ramus courses anteromedially, just lateral to the origin of the tensor tympani muscle, along the internal roof of the middle ear (Po3 108-03; Fig. 10C). Dorsal to the ectotympanic bone and posteroventral to the foramen ovale, it bifurcates into rami infraorbitalis and mandibularis (Po3 103-10; Fig. 10B). The ramus infraorbitalis courses posterior to CN-V3 in an anteromedial direction towards the ossified alisphenoid canal, via which it enters the cavum epiptericum to course ventral to the trigeminal ganglion (Po3 96-04). After exiting this space via the sphenorbital fissure, still below the main nerve bundle for CN V1 and V2, the ramus infraorbitalis gives off a laterally directed buccal arterial branch (Po3 76-01), and shortly thereafter a ventromedially directed dorsal palatine branch (Po3 73-10; Fig. 10A). During its anteriorly, medially, and ventrally directed course towards the palatine canal, the dorsal palatine artery gives off several small branches towards nearby tissues. Uniquely in this specimen, perhaps because of its larger size and more advanced ontogenetic age, a small sphenopalatine branch is evident (Po3 61-10, clearer on the right side). This branch leaves the dorsal palatine laterally, takes an indirect, dorsolateral path back up the palatine canal to then turn medially to enter the posteroventral margin of the nasal fossa (Po3 57-01), in a coronal plane overlapping the penultimate upper tooth (dP4).

Shortly before entering the ossified infraorbital canal, the ramus infraorbitalis gives off a large trunk that supplies a lacrimal branch and several smaller arteries that supply tissues in the orbital region, plus a branch leading dorsally into the diploë of the orbital wall, continuous with a large venous sinus between the eye and posterior ethmoidal region (Po3 64-04; Fig. 10A). Immediately distal to this orbito-lacrimal trunk, and also prior to entering the infraorbital canal, the ramus infraorbitalis supplies a superior alveolar artery (Po3 65-07; Fig. 10A) which enters a canal in the maxilla prior to the anterior termination of the bony infraorbital canal (Po3 58-04), and courses anteriorly within this canal supplying the upper toothrow. After exiting the infraorbital canal, the ramus infraorbitalis continues its anterior course and divides into several large branches to supply the densely packed sensory and muscular soft tissues of the anterior rostrum.

### Mandibular artery and nerve

After the bifurcation of the inferior stapedial artery, the ramus mandibularis courses laterally, dorsal to CN V3, towards the dentary which it enters via a mandibular foramen showing signs of ossification, but still defined medially by Meckel’s cartilage (Po3 90-04). Once inside the mandibular canal, the ramus mandibularis begins to regularly send small branches towards the developing teeth, with the first such branch coursing posteriorly and dorsally (Po3 84-06), just behind the posterior-most developing tooth (m1), still within its crypt. No dorsally directed foramina or spaces are evident in the ossified dentary beyond the alveoli dorsal to the developing teeth, all of which are still unerupted, and no major branches from the ramus mandibularis are evident beyond those supplying teeth and superficial tissues of the external jaw.

### The coronoid canal

**Figure 11:**
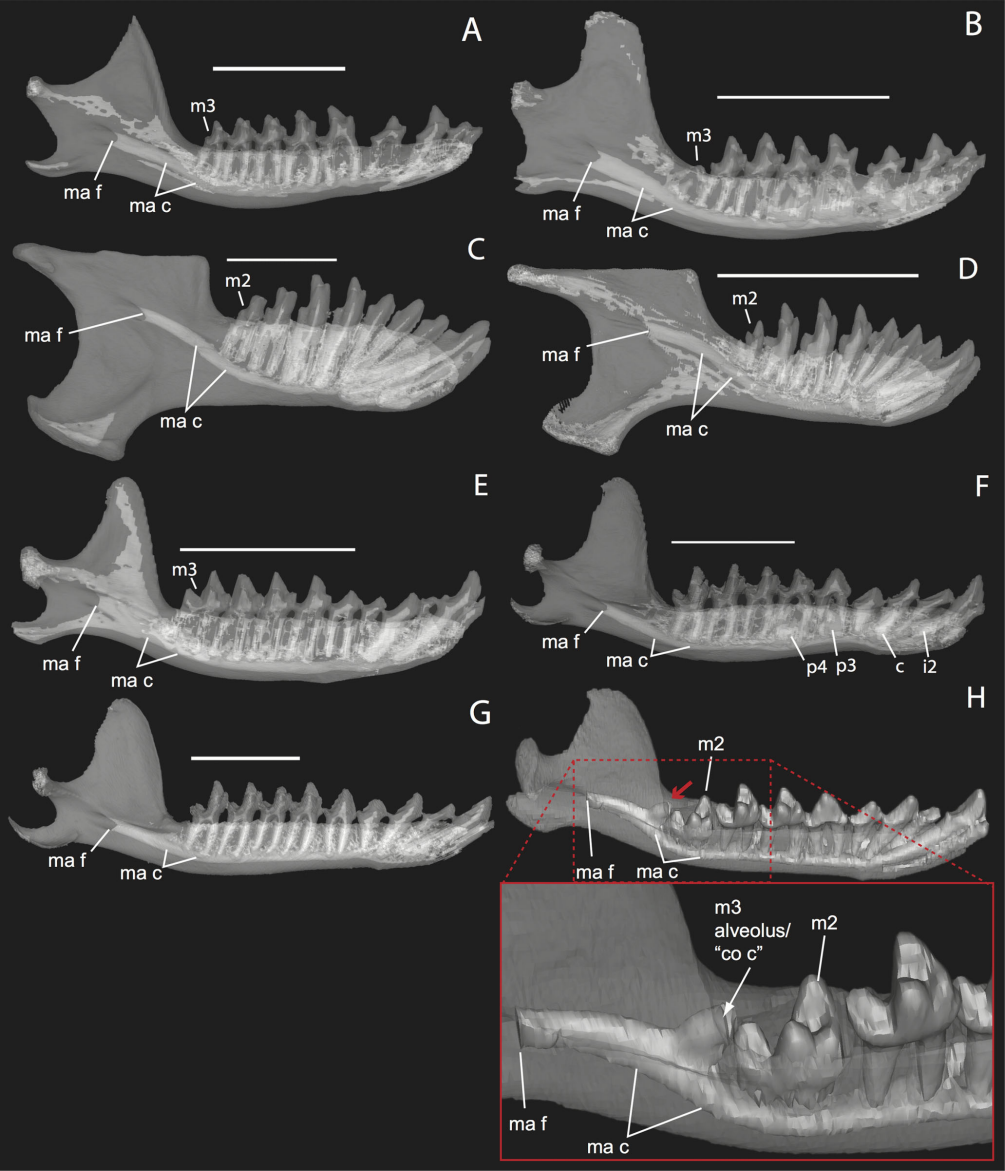
Lingual (internal) views of left jaws in *Setifer setosus* MNHN 1882-1566 (A) and MNHN 1962-1064 (B) *Amblysomus hottentotus* NFC 2 (C) and BMNH 4.6.6.3 (D) *Microgale dobsoni* MNHN 1932-3480 (E) *Potamogale velox*, UMZC E5425H (F) UMZC E5425I (G) and UMZC E5425B (H). Light shading represents sinuses, canals, and other internally open regions. For scanning details see Table 2; for abbreviations see Table 3. Scale bars (where available) indicate 1 cm, except (C and G) (5 mm).

**Figure 12:**
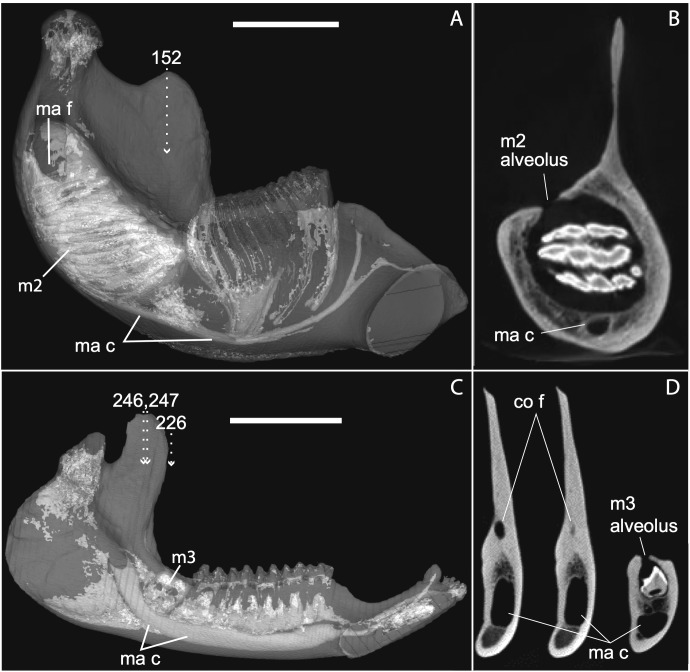
Medial views of jaws (A, C) and coronal sections of posterior alveolar regions (B, D) in *Elephas* (TMM M6445, (A, B)) and *Tapirus* (TMM M16, (C, D)). Dotted lines with white arrows represent approximate planes of coronal sections as numbered in stacks available at www.digimorph.org. The section in *Elephas* corresponds to the same individual and mirror-imaged μCT slice figured by Ferretti & DeBruyne (2011: their fig. 2G). Light shading represents sinuses, canals, and other internally open regions. Note the presence of a coronoid fossa (co f), but not foramen or canal, in *Tapirus*. Scale bars at the top of each image represent approximately 10 cm and are calculated based on values given at www.digimorph.org.

**Figure 13:**
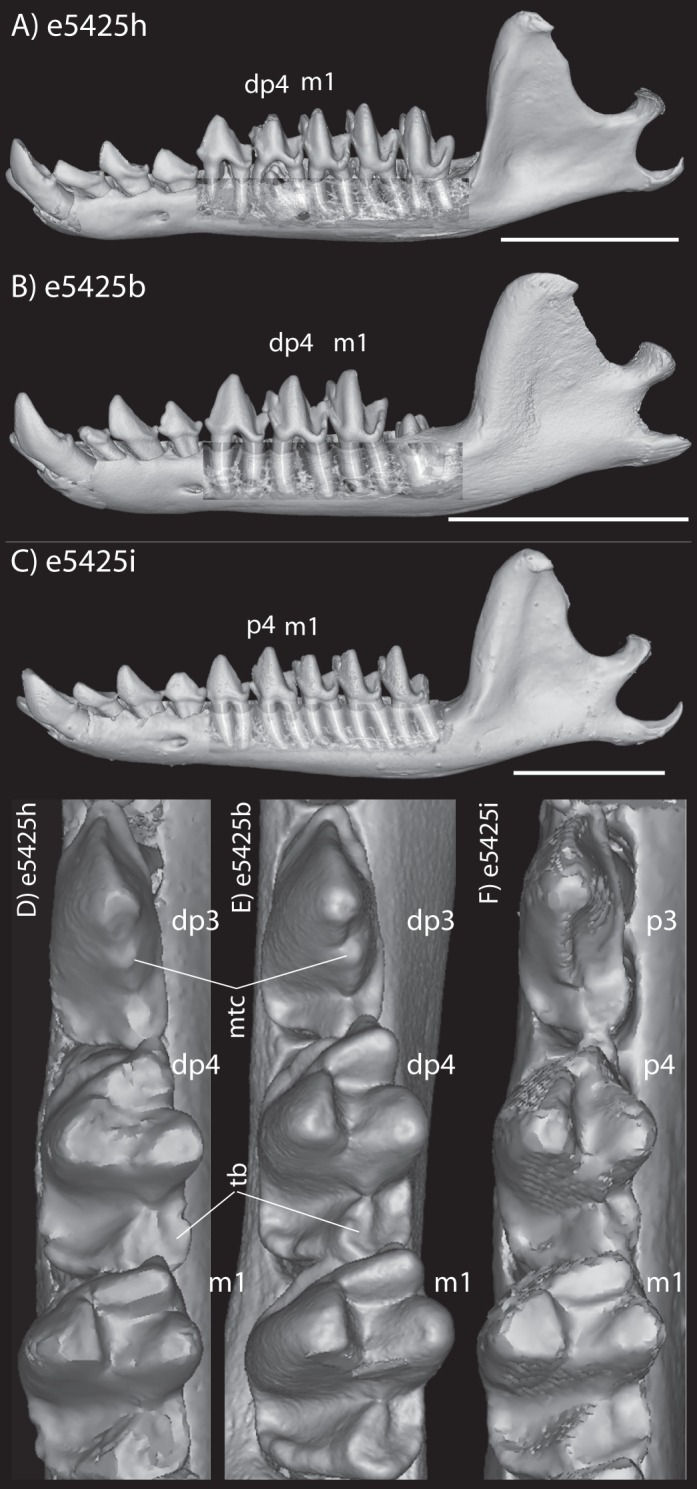
Lateral views of jaws of three individuals of *Potamogale velox*, UMZC E5425H (A, D) E5425B (B, E) and E5425I (C, F). The first two specimens lack a full, permanent dentition; in (C and F) the adult dentition is completely erupted. (D–F) show a closeup, occlusal view of the posterior-most two premolars and m1 for each specimen; note the metaconid (mtc) on dp3 and large talonid basin (tb) on dp4 (D, E) and lack thereof on p3 and p4 (F). UMZC E5425B shows its unerupted m2 in its alveolus (B) and we infer that it has not yet mineralized any of its permanent premolars or m3. Scale bars at bottom right of (A–C) represent 1 cm.

The embryonic specimens of *Potamogale* described above document several important features of their development and anatomy. However, our four histologically sampled individuals do not exhibit a true coronoid canal that is independent of posterior tooth alveoli (Figs. 1, 11 and 12). As noted in the introduction, this structure is consistently present in most adult paenungulates and has been reported in a fossil macroscelidid (Tabuce et al., 2012). We have observed a superficially similar structure in specimens of *Potamogale* (Fig. 11) and *Elephas* (Fig. 12) that are dentally immature. One *Potamogale* specimen (UMZC E5425B) shows a “canal” posterior to its still unerupted m2. This is a particularly interesting specimen; its antemolars are deciduous based on their crown and root morphology, for example in showing a paraconid on dp3, a relatively wide talonid basin on dp4, and widely spaced roots on dp3-4. However, it lacks calcified replacement teeth in the mandibular corpus (Fig. 13). Thus, the connection between mandibular canal and oral cavity in this specimen takes place via the somewhat abortive alveolus for the lower m3, which (as for the replacement premolars) completely lacks any sign of a developing tooth within it (Figs. 11H and 13). The lack of a mineralized p4 in this specimen is consistent with the fact that its m2 is largely in its crypt, the m3 is not yet mineralized either, and the reported eruption sequence for *Potamogale*, in which m3 erupts before the deciduous premolars are replaced (Asher & Lehmann, 2008; Asher & Olbricht, 2009). Hence, a patent coronoid canal is absent in all of the *Potamogale* specimens in our sample with a completely erupted dentition (e.g., BMNH 34.6.16.4, BMNH 30.11.11.187, BMNH 32.6.19.5, BMNH 98.10.7.6, BMNH 26.11.1.64, BMNH 19.5.8.17, BMNH 26.11.1.65, BMNH 26.11.1.63, UMZC E5425I).

## Discussion

### Development of cranial vasculature and the jaw in *Potamogale*

All of the embryonic specimens in our sample exhibit a carotid bifurcation, an occipital artery originating close to that bifurcation, supply of the lingual, facial, transverse facial, and superficial temporal arteries from the external carotid, posterior and small superior stapedial branches, an inferior stapedial that supplies rami mandibularis and infraorbitalis, and an internal carotid that supplies the Circle of Willis via the carotid foramen and ophthalmic arteries via the optic canal. With the exceptions of a reduced superior stapedial, this basic pattern has been generally understood to characterize the common ancestor of placental mammals and typifies most lipotyphlan and afrotherian “insectivorans” (Bugge, 1972; Cartmill & MacPhee, 1980; MacPhee, 1981; Asher, 2001).

Only our smallest specimen (Po2) lacked an obvious supply of the posterior auricular artery from the posterior stapedial, and branches for the deep cervical, ascending pharyngeal, recurrent laryngeal and superior thyroid. Five other anatomical differences among our embryonic specimens were particularly conspicuous, four vascular and one skeletal. Firstly, the smallest (Po2, HL 11 mm) and presumably youngest specimen shows the smallest superior stapedial ramus. This is more obvious in the other specimens, but still limited to supply of the meninges and temporal region. Unlike other insectivoran-grade species, *Potamogale* lacks an ossified sinus canal and supplies its ophthalmic artery via the internal-carotid supplied Circle of Willis, not superior stapedial ramus as in most other insectivoran-grade mammals (Asher, 2001). Secondly, our oldest specimen (Po3, HL 23 mm) shows greater development of what appear to be vascular heat-exchangers (Fig. 8), extending not only to branches of the occipital artery (as previously observed in *Potamogale*; Asher, 2001), but also to the ascending pharyngeal and deep cervical branches that become more abundant in the posterior and dorsal aspects of the neck (Fig. 10F). Thirdly, an obvious supply of the sphenopalatine artery from the ramus infraorbitalis is evident only in the largest of our three specimens, Po3. Arterial supply to the posterior nasal fossa is clearly necessary throughout development, but it is only in Po3 where an artery is evident piercing the ethmoid via a branch from the dorsal palatine in its course through the palatine canal (Fig. 10A). Fourthly, we observed variation in the origin of the superior thyroid artery. This vessel is not evident at all in our smallest specimen (Po2). It arises from the common carotid proximal to the origin of the external carotid in Po1 (Fig. 4), and arises from the external carotid near the origin of the recurrent laryngeal artery in our two largest specimens (Figs. 6 and 9). Finally, we observed increasing medial curvature of Meckel’s cartilage throughout development, prior to the detachment of the ear ossicles (see review in Maier & Ruf (2016)), corresponding to mediolateral growth of the mandibular condyle during development.

### Ossification sequence

Our four histologically preserved specimens indicate that the cranial ossification sequence in *Potamogale* is as follows: *Potamogale* first ossifies the bones around the periphery of the oral cavity, anterior braincase, and part of the squamosal (Po2). These are followed by ossifications in the pterygoids, jugal, and frontal (Po1), then the basicranium, vomer, palatines, and dermal braincase. Parts of the basisphenoid, occipital, petrosal, ethmoid, and mandibular condyles remain incompletely ossified in our largest specimen (Po3). Our overall sequence of alveolar oral cavity, face, basicranium, then posterior braincase is generally similar to those documented by Werneburg et al. (2013) for *Echinops* and *Tenrec,* although their sequences are not resolved to precisely the same degree.

### The coronoid canal

**Figure 14:**
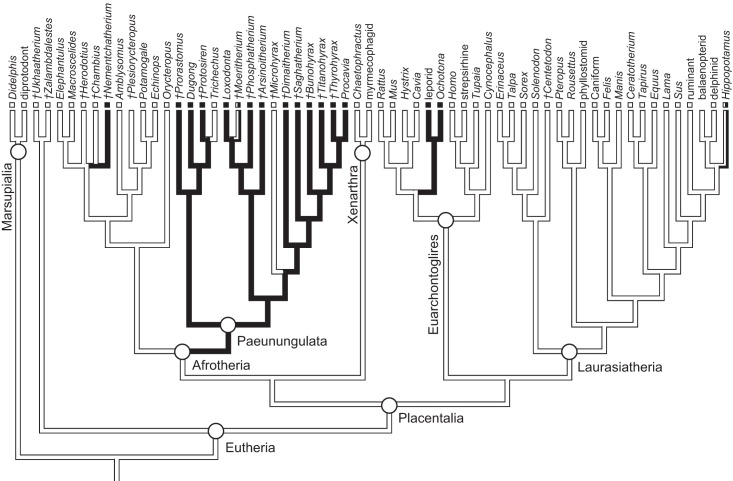
Phylogeny of eutherian mammals (rooted with two marsupials) derived from Meredith et al. (2011). Branch lengths are arbitrary. Positions of fossils (“†”) follow Asher (2007), except for *Plesiorycteropus* based on Buckley (2013), fossil proboscideans and macroscelideans based on Gheerbrant (2009) and Tabuce et al. (2012), hyracoids on Barrow, Seiffert & Simons (2012), and sirenians on Savage, Domning & Thewissen (1994) and Seiffert (2007). Black shading on branches indicates the parsimony-optimized presence of the coronoid canal; black and white squares at branch tips indicate presence or absence (respectively) of the coronoid canal in terminal taxa; absence of a square indicates uncertainty; dichromatic branches and squares indicate polymorphism.

As previously summarized, the coronoid canal is a rare feature in mammals. Reports in the literature (Tassy & Shoshani, 1988; Kondrashov, 1998; Meng, Hu & Li, 2003; Asher et al., 2005; Gheerbrant, Domning & Tassy, 2005; Ferretti & Debruyne, 2011) indicate its consistent appearance only among adult paenungulates and lagomorphs. We have also observed several specimens of *Hippopotamus* (but no other artiodactyl) in the collections of the UMZC with a coronoid canal. Figures 1, 11 and 12 show the morphology of the jaw and (when present) coronoid canal in μCT reconstructions of selected taxa. We map the distribution of this character in Fig. 14. As previously summarized, a patent coronoid canal, distinct from alveoli of posterior teeth, does not occur in *Potamogale*, appearing only among adult paenungulates and lagomorphs. Therefore, this character optimizes as a synapomorphy for paenungulates (Fig. 14), as hypothesized by previous authors (e.g., Tassy & Shoshani, 1988), and independently also for lagomorphs.

Ferretti & Debruyne (2011: figs. 2–4) interpreted a specimen of *Elephas maximus* (TMM-M6445, the same one figured here [Fig. 12] and available on www.digimorph.org) as possessing a coronoid foramen “merged with the alveolus of the distalmost erupting molar” (Ferretti & Debruyne, 2011: 398). However, we would argue that a structure anatomically equivalent to the coronoid canal they figure for two dentally mature proboscideans, the Eocene *Phosphatherium* and a modern *Loxodonta* (Ferretti & Debruyne, 2011: figs. 1 and 7E, respectively), is not present in TMM-M6445 (*Elephas*) or elephantines generally until the posterior-most lower molar becomes more completely erupted and a connection to the postero-internal aspect of the jaw opens behind the toothrow. What is evident in the TMM M6445 specimen of *Elephas* is simply the dorsal opening of the m2 alveolus. This is similar to the dorsal opening of the m3 alveolus in a specimen of *Tapirus* (TMM M16; Fig. 12), which also shows a fossa posterodorsally to the toothrow in the same position as the coronoid canal in, for example, *Procavia*, but which ends in a cul-de-sac. Certainty regarding the potential co-incidence of a coronoid canal with posterior tooth alveoli would require more postnatal specimens of elephantines showing the formation of the true coronoid canal (Ferretti & Debruyne, 2011: fig. 7E) during the course of dental eruption during postnatal ontogeny. We do not yet have access to such a series but welcome its application to this issue. We would however point out that ontogenetic variability may explain the polymorphisms coded in *Loxodonta* and *Moeritherium* by Seiffert (2007: character 280).

If the canal were present in taxa by virtue of dental alveoli, then every mammal with erupting teeth would exhibit a “coronoid canal,” as tooth eruption necessarily entails passage of the dentition through the mandibular corpus during postnatal development, and any alveolus opening above an erupting tooth would comprise a “canal” between the postero-internal aspect of the lower jaw and posterior toothrow (Figs. 1, 11 and 12) via their mandibular canals. Hence, the presence of an alveolus which in turn connects that space to the oral cavity is not, by itself, synonymous with the possession of a coronoid canal. Taxa with a coronoid canal, such as *Phosphatherium*, *Procavia* and adult elephantines, are distinct by virtue of the relations of this canal, which has its anterior opening posterior to the toothrow (Fig. 1).

Complicating this generalization is the condition present in manatees (*Trichechus*; Fig. 1C), which effectively lack a posterior terminus to their toothrow due to their ever-erupting supernumerary molars (Fig. 1C; see Domning & Hayek, 1984). Strictly speaking, then, they lack a bony canal connecting the space posterior to the toothrow with the postero-internal aspect of the lower jaw. We would nonetheless argue that the dentally immature specimen of *Elephas* figured here (Fig. 12) and in Ferretti & Debruyne (2011: figs. 2–4) shows only confluence between its m2 alveolus and the mandibular canal, and lacks a coronoid foramen or canal distinct from dental alveoli.

The coronoid canal was first identified as a synapomorphy of Paenungulates by Tassy & Shoshani (1988). Within Paenungulata, the coronoid canal is known in both living and extinct proboscideans, including basal taxa such as *Eritherium* and *Phosphatherium* (Delmer, 2005; Gheerbrant, 2009; Ferretti & Debruyne, 2011). It is also known in both living and extinct Sirenians (Savage, Domning & Thewissen, 1994) and most hyracoids (Barrow, Seiffert & Simons, 2010; Barrow, Seiffert & Simons, 2012; Tabuce et al., 2012). A coronoid canal has also been identified in the stem elephant shrew *Nementchatherium* (Tabuce et al., 2012). Our initial observations of a coronoid-canal-like structure in some specimens of *Potamogale* prompted us to investigate if this character might be more broadly distributed across afrotherian mammals (Fig. 14). In fact, it is not an afrotherian but a paenungulate synapomorphy, as previously hypothesized by Tassy & Shoshani (1988) and Gheerbrant, Domning & Tassy (2005). Importantly, the coronoid canal frequently occurs among stem paenungulates, including sirenians such as *Prorastomus* and *Protosiren* (Savage, Domning & Thewissen, 1994; Seiffert, 2007), proboscideans such as *Phosphatherium* (Gheerbrant, Domning & Tassy, 2005), embrithopods such as *Arsinoitherium* (Tabuce et al., 2012), and most hyracoids such as *Bunohyrax, Dimaitherium, Saghatherium, Titanohyrax,* and *Thyrohyrax* (Seiffert, 2007; Barrow, Seiffert & Simons, 2012; Tabuce et al., 2012), although interestingly not in *Microhyrax* (Barrow, Seiffert & Simons, 2010). Given the topology in Fig. 14 (Meredith et al., 2011), the coronoid canal forms a synapomorphy for paenungulates. On the other hand, due to the apparent lack of the canal in the currently basal-most hyracoid *Microhyrax* (Barrow, Seiffert & Simons, 2010), the optimization of the coronoid canal data as a paenungulate synapomorphy is ambiguous if hyracoids are reconstructed as the sister group of a sirenian-proboscidean clade (Asher, 2007; Gheerbrant, 2009).

## Conclusions

Our sample of a developmental series of the tenrecid afrothere *Potamogale* shows that, as expected, some aspects of its arterial supply change over the course of ontogeny. Variation in *Potamogale* includes the origin of the superior thyroid artery, the initial absence of the dorsal-palatine supplied sphenopalatine artery, and the increasing development of vascular plexuses in vessels supplying circulation in the back of the head, including occipital, ascending pharyngeal, and deep cervical arteries. We also observe an increase in posterior curvature of Meckel’s cartilage, corresponding with increased mediolateral size of the mandibular condyle.

The presence of spaces in the dentary connecting the oral cavity to the internal aspect of the jaw fluctuates during ontogeny, and connections will exist between the mandibular canal, internal aspect of the coronoid process, and tooth alveoli in any developing mammal. However, the developmental anatomy of *Potamogale* shows that fully adult specimens lack a coronoid canal. Although small nutrient foramina may join the posterior alveolar region to the mandibular canal and internal coronoid region in many different species, a large, consistently developed coronoid canal is present only among dentally mature individuals of paenungulates, some fossil macroscelidids, lagomorphs, and possibly hippos, opening at the base of the coronoid process, posterior to the last lower tooth. Optimized on a well-corroborated phylogeny of placental mammals, a large, patent coronoid canal is an independent synapomorphy for two clades: Paenungulata (proboscideans, sirenians, hyracoids) and Lagomorpha (ochotonids, leporids). Interestingly, placing proboscideans and sirenians together in Tethytheria (as in Tassy & Shoshani (1988) and Asher (2007)) results in an ambiguous optimization of the coronoid canal. This is due to the apparent lack of the canal in *Microhyrax* and its basal position within Hyracoidea. According to Seiffert (2007) and Barrow, Seiffert & Simons (2010), this taxon lacks a coronoid canal, although Tabuce et al. (2012: 953) state that it is present. We suspect that, as in proboscideans (including fossils such as *Moeritherium*), there is ontogenetic variation that complicates character assessment in fossils such as *Microhyrax* which generally lack detailed ontogenetic series. In any event, among afrotherians, a patent coronoid canal is clearly widespread among living and fossil hyracoids, sirenians, and proboscideans, and it occurs in at least in one fossil macroscelidid (*Nemchetatherium,* Tabuce et al., 2012). Although we do not find strong evidence that the coronoid canal is a shared feature of afrotherians, we nonetheless agree with previous authors (Tassy & Shoshani, 1988) that it is likely a paenungulate synapomorphy. A focus on the soft-tissue contents of this canal of these groups, and on the postnatal osteological changes to the lower jaw in taxa with a coronoid canal and dental eruption well into adulthood (e.g., proboscideans and hyracoids) are logical, additional steps to further investigate this structure.

## Additional Information and Declarations

### Data Deposition

The following information was supplied regarding data availability: Our raw data are conveyed in the figures, tables, and text comprising this manuscript.
